# Longitudinal live-cell chemical imaging using coherent Raman scattering microscopy

**DOI:** 10.1039/d6an00802j

**Published:** 2026-09-17

**Authors:** Chi Zhang, Seohee Ma, Mingmin Zhou, Laura L. Lukov, Nikta Zafarjafarzadeh

**Affiliations:** a Department of Chemistry, Purdue University 560 Oval Dr. West Lafayette IN 47907 USA zhan2017@purdue.edu; b Purdue Institute for Cancer Research 201 S. University St. West Lafayette IN 47907 USA; c Purdue Institute of Inflammation, Immunology, and Infectious Disease 207 S. Martin Jischke Dr. West Lafayette IN 47907 USA

## Abstract

Optical methods enable minimally invasive analysis of chemical distributions and dynamics in biological specimens. Many optical imaging modalities have been developed to generate chemical contrast based on intrinsic molecular signatures or highly specific labeling. Coherent Raman scattering (CRS) microscopy has emerged as a powerful label-free technique for imaging biomolecules or exogenous compounds in biological samples. Most CRS imaging studies have focused on extracting chemical distribution, composition, and abundance in samples, and work in these areas has been extensively reviewed. However, an important aspect of chemical processes—the dynamics of chemical changes over time—has received less emphasis in CRS microscopy. Many critical insights into chemical processes lie in the spatiotemporal evolution of molecular species in live samples. Such information can only be obtained through live-cell imaging, and more importantly, longitudinal imaging of the same sample over time. In this review, we focus on key parameters related to longitudinal live-cell CRS microscopy and major recent studies. We first discuss three primary factors that must be considered for longitudinal CRS imaging: limit of detection, chemical selectivity, and phototoxicity. Limit of detection and chemical selectivity determine what CRS can measure, whereas phototoxicity dictates whether longitudinal imaging can capture meaningful cellular chemical dynamics with minimal perturbation to biological functions. In these discussions, we also provide critical comparisons between CRS microscopy and fluorescence microscopy. We then review recent developments and achievements in longitudinal CRS microscopy. Finally, we provide perspectives on future directions and areas where longitudinal CRS microscopy can make contributions to advancing our understanding of biological processes.

## Abstract 2

CRS microscopy enables label-free and minimally invasive imaging of chemical dynamics. This review discusses detection limits, chemical selectivity, phototoxicity, recent advances, and future directions of longitudinal CRS microscopy in live cells.

## Graphical abstract

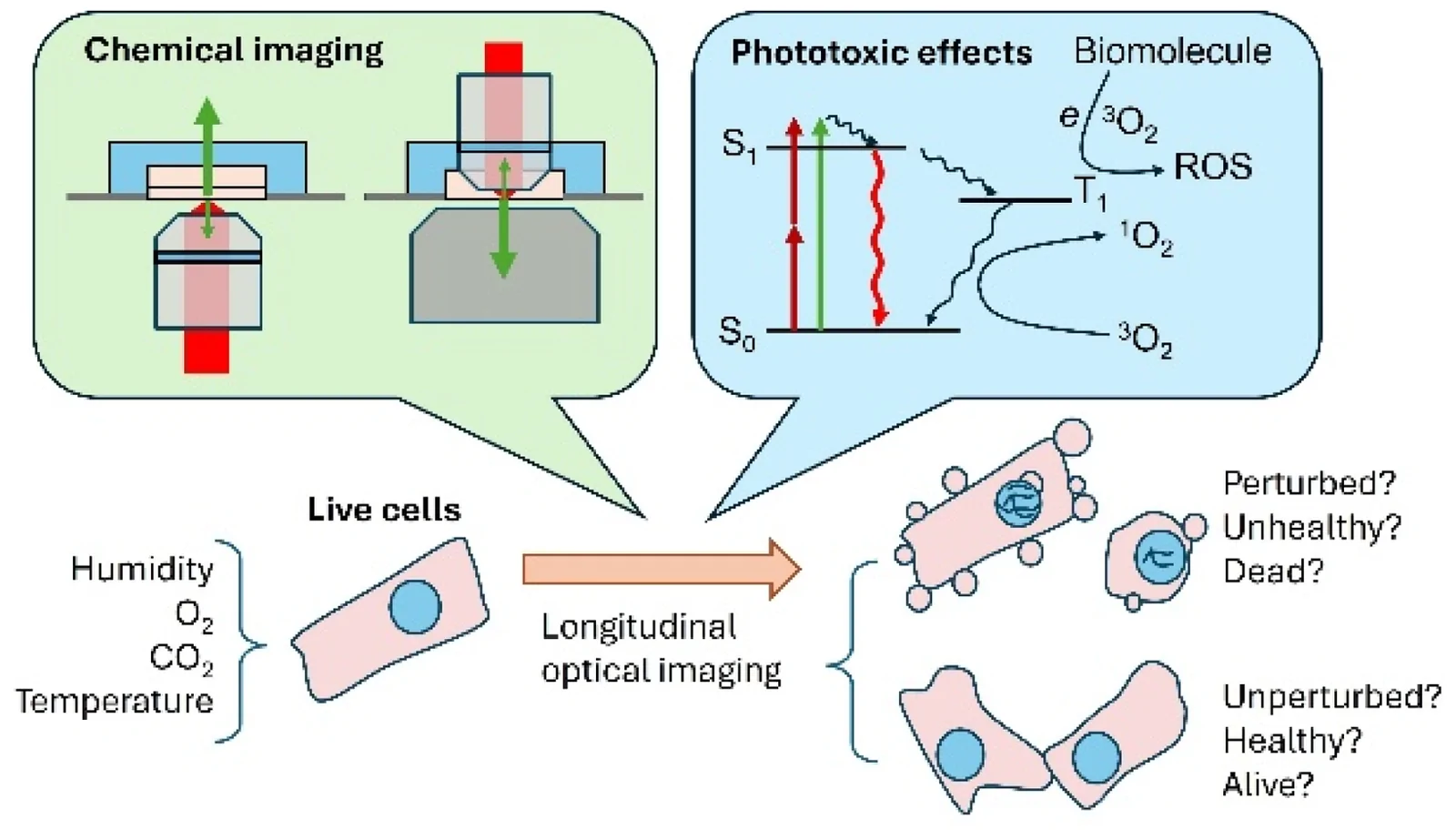

## 1. Introduction

Biological organisms are intricate assemblies of molecules that determine their structures and functions. The identity, abundance, spatial distribution, interactions, and dynamics of these biomolecules collectively constitute the chemical information that drives and regulates biological processes. Obtaining chemical information in live organisms is critical for understanding the fundamental mechanisms of life and disease transitions. Various strategies have been developed to extract chemical information from complex biological systems. Among these approaches, optical imaging is one of the most widely used and powerful categories. Compared with many other chemical imaging modalities, a key advantage of optical imaging is minimal invasiveness combined with high spatial resolution. Photons can be delivered precisely to locations of interest with penetration depths from micrometers to millimeters, while chemical information is extracted through various light–matter interactions. Optical imaging is particularly well-suited for imaging organisms in their native state, which is critical for live-cell analysis, especially for longitudinal studies.

Live cells are highly dynamic systems whose molecular composition and functional states change continuously over time. Although static images from fixed samples can provide valuable information, they offer only limited insight into how cellular behaviors change over time. Many important biological processes, such as protein dynamics, stress responses, cell-cycle progression, and metabolic remodeling, involve coordinated changes that occur across both space and time and can only be extracted from time-dependent changes in the same biological specimens. Therefore, longitudinal chemical imaging of live cells is critically important.

Among optical imaging methods, fluorescence microscopy is the most widely used technique for chemical imaging. Fluorescence microscopy has been a central optical tool for live-cell chemical imaging because it enables specific biomolecules and subcellular structures to be visualized with high sensitivity and spatial resolution. Its chemical selectivity largely arises from powerful labeling strategies that allow highly specific targeting of biomolecules, especially proteins and nucleic acids.^1,2^ A variety of fluorescence probes is also available for imaging metabolites. Additionally, fluorescence imaging offers an exceptionally good limit of detection (LOD), enabling the detection of extremely low chemical contents and even single-molecule activities in complex biological environments.

Despite these advantages, fluorescence labeling using organic dyes or antibodies has the limitation of requiring the preparation and delivery of fluorescent probes, which must cross the cell membrane and selectively bind to the target of interest. Genetically encoded fluorescent reporters have largely addressed this limitation by enabling cells to express fluorescent proteins internally. However, their use generally requires genetic transfection or other forms of genetic manipulation, which alters the genetic information of the cells. Furthermore, fluorescent molecules often suffer from photobleaching and can fade over time. Their selectivity is often limited when targeting metabolites or small molecules, and the relatively large fluorophore structure can perturb the function of the labeled molecule. Moreover, excited fluorophores may lead to phototoxicity, potentially altering biomolecular functions and cellular behavior.^3^

These limitations have led to growing interest in label-free imaging techniques that allow biomolecules to be identified without adding external probes. To obtain chemical information without labeling, one powerful approach is to utilize molecular vibrational signatures. Such information is typically assessed through mid-infrared (mid-IR) absorption or Raman scattering. Vibrational imaging methods can provide high chemical selectivity for small molecules and metabolites.^4,5^ They can also be combined with exogenous vibrational tags, which are usually smaller than fluorescent tags, produce sharper spectral features, and exhibit lower phototoxicity.^6,7^

For biological samples, the delivery of mid-IR light deeper into the sample is challenging due to strong water absorption in the mid-IR frequency region. Raman scattering, on the other hand, typically uses visible or near-IR light, which experiences much weaker water absorption and therefore is more suitable for imaging biological specimens in their native environments. Nevertheless, spontaneous Raman scattering is intrinsically inefficient and requires a longer signal integration time to accumulate enough photons. This limitation makes live-cell imaging and high-speed quantification of dynamic biological processes extremely challenging. Although line-scan Raman microscopy can improve imaging speed,^8,9^ it still lags significantly behind the typical speed of fluorescence imaging. Several methods have been developed to enhance Raman scattering efficiency, such as resonance enhancement,^10^ surface-enhanced Raman scattering (SERS),^11,12^ and coherent Raman scattering (CRS).^13–15^ Among these methods, CRS is best suited for high-speed far-field biological imaging in both the label-free and the labeling-enhanced manner.

Two major CRS modalities are widely used for biological imaging: coherent anti-Stokes Raman scattering (CARS) and stimulated Raman scattering (SRS).^13–15^ Typically, near-IR picosecond or femtosecond pulses are employed to minimize phototoxicity and optimize laser penetration depth. Both CARS and SRS provide sufficient photon budget for high-speed imaging of chemical compositions that have enough concentration. These methods have been widely applied to visualize neutral lipids, total proteins, chromosomes, drug compounds, and other metabolites in cells and larger organisms. The high imaging speed of CRS techniques also enables the quantification of the dynamics of chemical compartments in live cells. Such dynamic information serves as an important readout of cellular functions and mechanisms that cannot be extracted from static imaging or fixed samples.

The principles and technological advances of CRS techniques, as well as CRS applications in biological imaging, have been extensively reviewed in previous publications.^14,16–23^ However, relatively little attention has been devoted to longitudinal live-cell imaging and phototoxicity in CRS microscopy. Longitudinal imaging refers to continuous or repeated imaging of the same field of view (FOV) over an extended duration to track temporal changes in the same population of cells. In this review, we first discuss three critical factors that should be considered for longitudinal CRS microscopy: limit of detection (LOD), chemical selectivity, and phototoxicity. In these discussions, comparisons with fluorescence microscopy are also included. We then review the progress made in applying CRS microscopy to longitudinal live-cell imaging. Finally, we provide perspectives on future trends in live-cell studies enabled by longitudinal CRS imaging.

## 2. Limit of detection of CRS microscopy

The LOD of CRS microscopy is typically much higher (inferior) than that of fluorescence microscopy. This difference arises from the fundamental physical values of electronic absorption and Raman scattering cross sections. Electronic absorption is roughly 10^12^–10^14^ times stronger than Raman scattering.^24^ Given the high quantum yields of many fluorophores, fluorescence emission from a fluorophore can also be trillions of times stronger than its Raman scattering signals.^24^

**Fig. 1:**
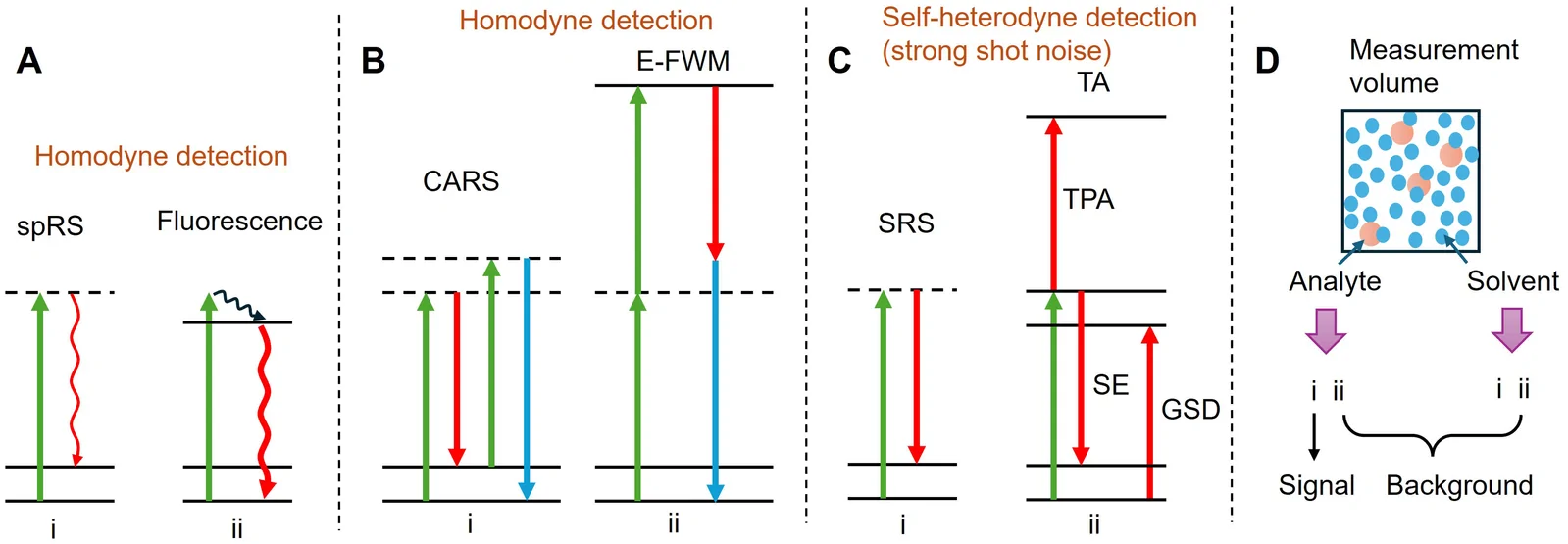
Signals and backgrounds in spontaneous and coherent Raman scattering. (A) Energy diagram of spontaneous Raman scattering (spRS, i) and fluorescence emission (ii). (B) Energy diagram of coherent anti-Stokes Raman scattering (CARS, i) and electronic-state-mediated four-wave mixing (E-FWM, ii). (C) Energy diagram of stimulated Raman scattering (SRS, i) and transient absorption (TA, ii) at zero time delay. Two-photon absorption (TPA), stimulated emission (SE), and ground state depletion (GSD) are three optical processes involved in TA. (D) An illustration of analytes in a solution, the simplest model of biological chemical imaging. The mechanism i from the analyte contributes to Raman signals, while mechanisms i and ii from the solvent and mechanism ii from the analyte contribute to the background. The detection limit of the system can be strongly affected by the background.

In the same environment, fluorescence and Stokes Raman scattering can act as backgrounds to each other (Fig. 1A). Because fluorescence emissions from organic dyes or fluorescent proteins are typically orders of magnitude stronger than Raman scattering, the Raman background is usually negligible for fluorescence measurements, except in specialized scenarios such as single-molecule detection.^25,26^ In contrast, fluorescence background in Raman spectroscopy can be devastating.^27,28^ Strong fluorescence emissions can completely overshadow Raman features by introducing enormous shot noise.

In CRS microscopy, the fluorescence background is largely suppressed. However, CRS techniques are not completely background-free. CARS is typically accompanied by a strong nonresonant background originating from both analytes and solvents, especially when electronic-state-mediated four-wave mixing is strong (Fig. 1B).^29,30^ SRS can exhibit background signals from strong light absorbers that have transient absorption (Fig. 1C).^19,31^ The light absorption can also result in a local temperature rise that could contribute to the photothermal background. Even in the absence of transient absorption, SRS has a relatively high shot-noise floor due to the direct detection of the intense laser source (self-heterodyne detection). Furthermore, the water background presents a major challenge for all Raman microscopy techniques (Fig. 1D).^32^ Water is not a spectroscopically silent solvent in Raman scattering, and its extensive hydrogen-bond network leads to extremely broad Raman bands. The resonant and nonresonant responses from water span almost the entire Raman spectrum. The ratio between the resonant response of analytes and the nonresonant response of water is usually much lower than the fluorescence-to-Raman ratio (which can reach the trillion level). As a result, the practical LOD of Raman methods is typically in the order of tens to hundreds of thousands of C–H bonds (or millimolar to micromolar) in water.^33–35^ Moreover, this value is generally obtained by averaging spectra from thousands of pixels acquired over a total time scale ranging from hundreds of milliseconds to tens of seconds. During imaging, the actual LOD at each pixel is typically much lower.^33^

To improve LOD, Raman tags can be introduced to enhance resonant responses or shift signals into spectrally silent regions (with lower water background).^36,37^ However, even with such strategies, the achievable LOD is still generally not comparable to that of most fluorophores in fluorescence microscopy. Although techniques such as SERS or CRS have been demonstrated to detect single molecules in special cases,^38–40^ usually when background Raman signals are sufficiently suppressed by nanostructure local enhancement^37,38^ or the Raman bonds are conjugated with fluorescence detection,^40^ these approaches are typically not practical for routine far-field label-free biological imaging or noninvasive live-cell analysis.

In summary, the most practical LOD for intrinsic biomolecules in CRS microscopy is typically in the millimolar to micromolar range in water (*e.g.*, C–H bonds),^33–35^ which is sufficient for imaging lipid droplets (LDs), overall cellular protein content, high-concentration glucose, enriched vitamin A, and similar targets.^41–44^ Detecting intrinsic biomolecules in the low micromolar or nanomolar range is generally not feasible with conventional Raman microscopy. For specially designed Raman tags, the LOD can sometimes reach the micromolar or nanomolar range.^40,45,46^ In contrast, fluorescence imaging techniques have the lowest LODs in aqueous environments among all analytical methods, with nanomolar to femtomolar detection, and even single-molecule sensitivity, being routinely achievable. A summary of major noise factors, background contributions, and LOD for fluorescence and Raman techniques is provided in Table 1.

**Table 1:** Comparing major noise sources, background contributions, and LOD of fluorescence and Raman microscopy techniques

	Fluorescence	Raman		
		Spontaneous	CARS	SRS
Noise (major)	• Detector dark current shot noise	• Detector dark current shot noise	• Detector dark current shot noise	• Laser shot noise
	• Electronic noise	• Electronic noise	• Electronic noise	• Laser intensity 1/*f* noise
Background	• Raman (extremely low)	• Fluorescence (when present)	• Non-resonant background	• Transient absorption (strong light absorbers)
	• Autofluorescence	• Water	• Water	• Photothermal (strong light absorbers)
				• Water
Typical LOD (in water)	• nM–fM; single molecule	• Sub-mM (C–H)	• Sub-mM (C–H)	• Sub-mM (C–H)

## 3. Chemical selectivity of CRS microscopy

**Fig. 2:**
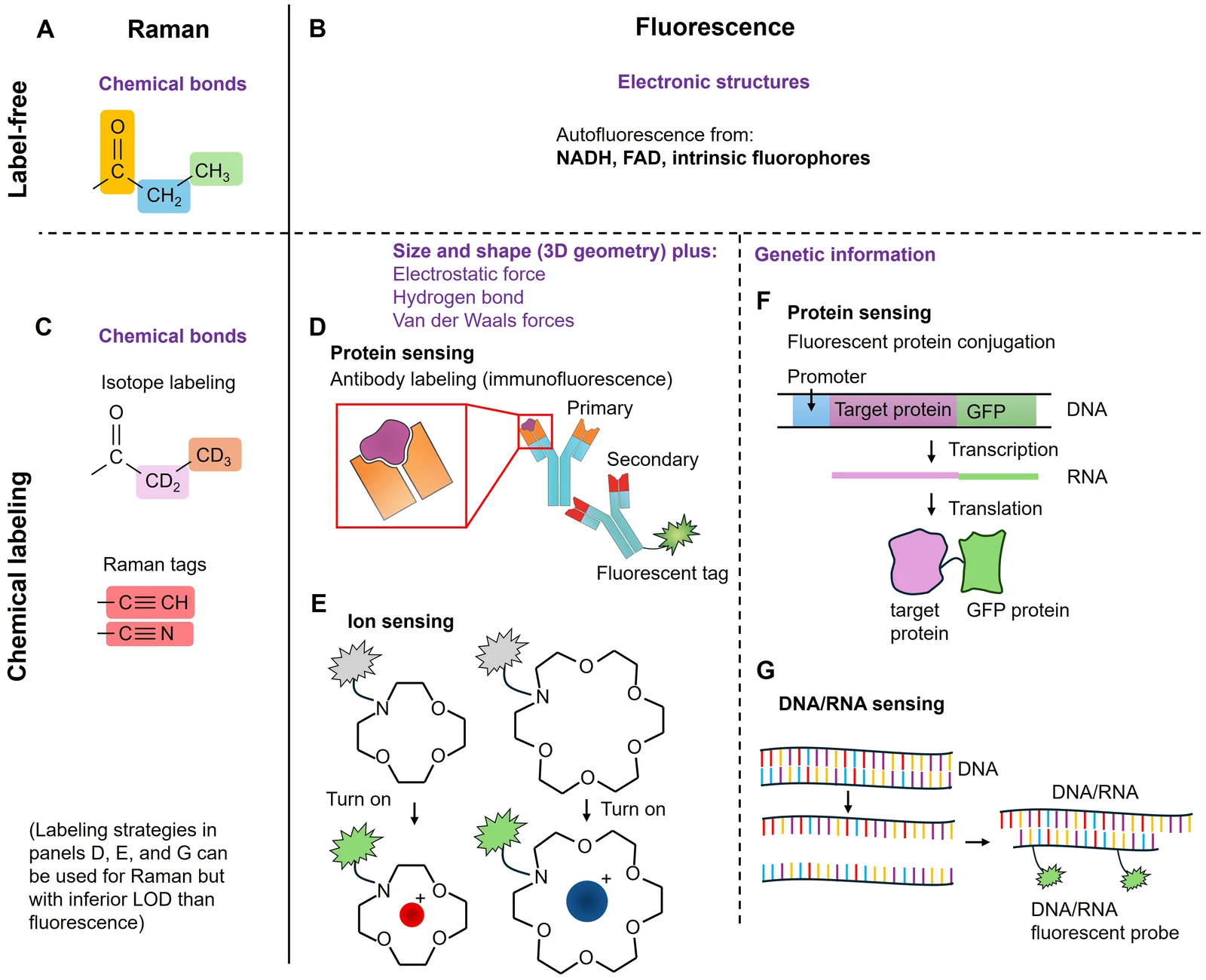
Illustrations of chemical selectivity in Raman and fluorescence imaging. (A) Label-free Raman detection, where chemical selectivity arises from intrinsic vibrational signatures of chemical bonds. (B) Label-free fluorescence detection of intrinsic autofluorescent molecules, with selectivity determined by electronic structures. (C) Raman-based chemical labeling using isotope substitution and Raman tags to introduce distinct vibrational signatures. (D) Protein detection *via* immunofluorescence relies on highly specific antibody-epitope interactions. (E) Fluorescent turn-on ion sensing using crown ether-based probes with selective host–guest binding. (F) Fluorescent protein tagging through genetic transfection for target-specific labeling. (G) DNA/RNA detection using sequence-specific fluorescent probes. Chemical selectivity in panels A and C originates from chemical bond vibrations; in B from electronic structure; in D and E from size- and shape-dependent molecular recognition and specific intermolecular interactions; and in F and G from genetically encoded or sequence-defined specificity.

Different from fluorescence microscopy, in which chemical selectivity is largely determined by various labeling strategies, the chemical selectivity of Raman spectroscopy, including CRS, primarily arises from the detection of molecular vibrational signatures associated with specific chemical bonds (Fig. 2A). This characteristic means that Raman spectra typically possess higher intrinsic chemical specificity than fluorescence spectra (Fig. 2B).^47,48^ However, such intrinsic chemical selectivity, compared to chemical labeling, is generally more effective for small molecules than for complex macromolecules. Isotope labeling and Raman tags can also be used to enhance chemical selectivity (Fig. 2C),^40,45,46,49^ though these techniques are predominantly used for tracing metabolites or generic classes of proteins and nucleic acids.

Biological samples are typically complex mixtures, and successful Raman detection requires that the molecular targets reach sufficient concentration and exhibit distinctive chemical bond signatures compared with surrounding molecules. In mammalian cells, neutral lipids, due to their hydrophobic nature, tend to aggregate into densely packed organelles called lipid droplets (LDs).^50,51^ These LDs are primarily composed of triglycerides and cholesteryl esters, which are rich in CH_2_ functional groups. As a result, they typically generate the strongest signals in the CH-stretching vibrational region. Hyperspectral CRS microscopy can further improve the separation of LDs from other cellular components and can even reveal compositional changes in neutral lipids within the droplets.^52–54^ Unlike mass spectrometry, CRS generally cannot resolve detailed lipid structures such as specific lipid chain species or precise unsaturation levels.^55,56^ However, it can provide useful information about the overall variations in cholesteryl ester levels, lipid saturation, and average lipid chain length.^57–60^

In addition to LDs, other small molecules, such as drugs accumulated within cells,^61^ can be detected by targeting their characteristic Raman peaks. However, the intracellular concentration of these molecules must have a high enough enrichment for confident detection. Other enriched exogenous or intrinsic molecules that have sufficient concentrations and distinctive spectral signatures, such as polymer microparticles, can also be effectively measured using CRS microscopy in cells.^62^ Certain endogenous molecules can also be detected under favorable conditions. For example, vitamin A can be observed in LDs within liver cells, and glycogen can be detected in specific cell types or tissues.^41,42^ Cytochromes, which are often detectable in spontaneous Raman scattering microscopy due to resonance enhancement,^63,64^ are difficult to detect using near-infrared-based CRS microscopy.

Large biomolecules such as proteins and DNAs are composed of many similar chemical bonds, making their Raman spectra generally insufficient to distinguish individual protein species in most cases, unless they are extremely enriched and possess distinctive spatial structures. However, Raman microscopy can still provide useful information about the total protein concentration within cells or subcellular compartments.^65–67^

In contrast, fluorescence microscopy offers exceptional molecular specificity for proteins through highly selective labeling strategies. One major mechanism underlying this specificity is binding affinity determined by the size and shape complementarity between the sensor and its molecular target. For instance, fluorescently conjugated antibodies achieve high specificity by binding to defined epitopes (Fig. 2D),^68,69^ while fluorescent crown ether-based turn-on sensors selectively detect ions through precise host–guest interactions within their binding pockets (Fig. 2E).^70^ Beyond geometric-based recognition, high chemical selectivity can also be encoded genetically. Examples include the expression of fluorescent proteins fused to specific target proteins (Fig. 2F),^71–73^ as well as highly selective DNA and RNA detection using predesigned probes with complementary sequences (Fig. 2G).^74–78^ These labeling approaches typically generate selective and strong fluorescence signals that can be easily distinguished from cellular autofluorescence, enabling sensitive and specific visualization of targeted proteins, ions, or DNA/RNA molecules. In addition, a wide range of fluorescent probes have been developed to detect metabolites that are generally beyond the reach of Raman-based methods in cellular systems, such as H_2_O_2_, ATP, and GTP.^79–81^

Raman-based labeling strategies have also been developed using chemical tags such as alkyne, cyano, and more conjugated structures that exhibit strong Raman scattering cross-sections and minimal overlap with endogenous biomolecular signals (Fig. 2C).^36,82–84^ These Raman tags are much smaller than fluorescent labels and are considered bioorthogonal because such vibrational signatures are rare in biological systems.^85^ However, the detection sensitivity of Raman-based methods remains significantly lower than that of fluorescence microscopy. Consequently, although Raman tags show strong potential for multiplexed imaging^86,87^ and can also be used with antibody conjugation, their LOD currently cannot compete with that of fluorescence techniques. Furthermore, due to the bioorthogonal chemical nature of these tags, they are difficult to genetically encode into proteins,^88^ which prevents them from being co-expressed with proteins of interest through standard genetic transfection approaches.

In summary, Raman-based imaging is generally more suitable for detecting small molecules present at relatively high concentrations, whereas fluorescence microscopy is more suitable for imaging proteins, nucleic acids, and ions due to highly specific labeling strategies and extremely low LOD. For these reasons, Raman techniques typically serve a complementary role rather than a central optical technology in biological research. A summary of the chemical selectivity of fluorescence *versus* Raman imaging techniques is provided in Table 2.

**Table 2:** Comparison of fluorescence and Raman techniques for chemical selectivity in label-free and labeled modes for imaging cells. Chemical selectivity levels (low to high) are indicated in parentheses

	Fluorescence	Raman		
		Spontaneous	CARS	SRS
Label-free	• Usually nicotinamide adenine dinucleotide hydrogen (NADH), flavin adenine dinucleotide (FAD), and special pigments (Low)	• Enriched small molecules such as lipids, retinol, drug compounds, and water (High)		
		• Certain exogenous or endogenous polymeric substances, such as glycogen, microplastics (High)		
		• Proteins (Low)		
		• DNA/RNA (Low)		
Labeling-enhanced	• Proteins (High, *via* antibody labeling, genetically encoded fluorescent proteins)	• Isotope labels: metabolites (High); proteins (Low, higher *via* antibody labeling)		
	• DNA/RNA (High)	• Raman tags: metabolites (High); proteins (Low, higher *via* antibody labeling)		
	• Metabolites (low to high); lipids (Low)	(Typically, LOD is far poorer than fluorescence techniques)		
	• Ions (High)			

## 4. Phototoxicity and photoperturbation in longitudinal CRS microscopy

This section discusses the mechanisms of phototoxicity and photoperturbation in optical microscopy, especially CRS; outlines the theoretical framework of photoperturbation and SNR in CRS; analyzes key experimental factors influencing phototoxic effects; summarizes established metrics for quantitatively assessing photodamage in live cells; and provides some practical guidelines for minimizing photodamage during live-cell imaging.

### 4.1 Mechanisms of phototoxicity and photoperturbation

**Fig. 3:**
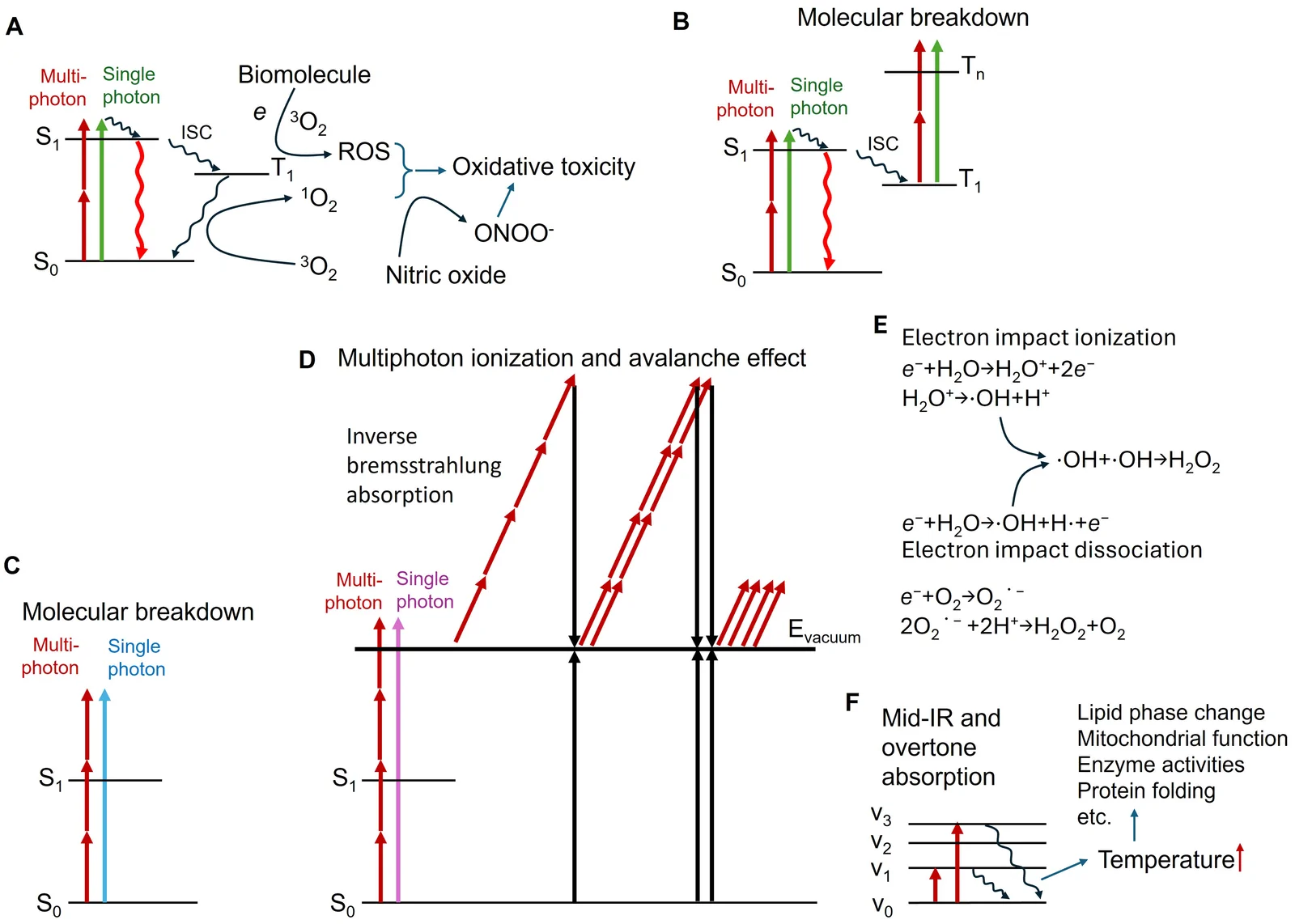
Phototoxicity and photoperturbation mechanisms in various optical imaging modalities. (A) Single- or multiphoton excitation promotes biomolecules to singlet excited states, which can undergo intersystem crossing (ISC) to populate triplet states. These excited states can subsequently generate singlet oxygen and other reactive oxygen and nitrogen species, leading to oxidative damage. (B) Triplet-state-absorption-mediated molecular degradation. (C) Direct molecular breakdown induced by single- or multiphoton absorption. (D) Photoionization processes, including single- and multiphoton ionization, as well as avalanche ionization driven by inverse bremsstrahlung absorption. (E) High-energy free electrons interact with water and oxygen molecules, resulting in the production of reactive oxygen species (ROS). (F) Mid-infrared fundamental and overtone absorption of molecular vibrations. Local temperature change could impact lipid phase, mitochondrial functions, enzyme activities, protein folding, and more. Thermal effects are involved in all these processes, with the temperature rise associated with laser dose and the absorption cross-sections of the involved molecules. S: singlet state; T: triplet state; v: vibrational state; *E*_vacuum_: energy of a free electron in vacuum.

One important factor in optical imaging, particularly for longitudinal live-cell studies, is phototoxicity, as photons can induce both direct and indirect perturbations to biological systems, leading to cellular damage. The most common source of phototoxicity in visible and near-IR imaging is the generation of ROS (Fig. 3A).^89,90^ This process typically involves molecules with strong spin–orbit coupling that undergo intersystem crossing to long-lived triplet states following single- or multiphoton absorption (Fig. 3A). These triplet states can subsequently interact with surrounding biomolecules or molecular oxygen to produce ROS and reactive nitrogen species (RNS),^90,91^ resulting in oxidative stress. Many fluorescent molecules exhibit such photosensitizing effects upon excitation, making phototoxicity a critical concern in long-term live-cell imaging.^3,92^ In these cases, molecular oxygen plays a central role as a precursor to singlet oxygen and other ROS. Genetically encoded fluorescent proteins can exhibit lower phototoxicity than exogenous fluorescent dyes. One contributing factor is that the β-barrel in many fluorescent proteins physically shields the chromophore and limits its access to molecular oxygen, thereby reducing light-induced ROS generation.^93,94^ In addition, fluorescent proteins can be expressed at relatively low concentrations and localized specifically to the structures or molecules of interest, reducing nonspecific labeling and the need for high concentrations of exogenous fluorophores. These features can contribute to lower phototoxicity while maintaining comparable fluorescence performance. However, exceptions exist. For example, KillerRed exhibits strong light-induced phototoxicity, which has been associated with a water-filled channel extending through the β-barrel toward the chromophore, facilitating access of oxygen and other molecules to the chromophore.^95^ ROS damage biomolecules through oxidative reactions with lipids, proteins, and nucleic acids, leading to peroxidation of lipids and proteins, and DNA damage.^96^ In addition, triplet-state absorption can further promote molecular degradation, inducing direct damage to endogenous biomolecules or generating toxic byproducts that interfere with cellular functions (Fig. 3B).^97,98^

Direct photon-induced molecular damage is typically significant only at high photon energies, such as under ultraviolet (UVA-UVC) illumination or *via* multiphoton absorption.^99–103^ These pathways can disrupt biomolecules without requiring triplet-state formation or ROS intermediates.

CRS microscopy typically employs high-intensity pulsed lasers in the near-IR range, which can still induce significant photodamage in live cells and tissues. Although linear (single-photon) absorption is reduced at these wavelengths, ultrafast pulses can drive nonlinear excitation of both intrinsic and exogenous molecules through multiphoton processes, as illustrated in Fig. 3A–C.^103^

At sufficiently high peak powers, laser pulses may also induce multiphoton ionization, generating low-density plasma and hot electrons,^104,105^ and triggering avalanche ionization *via* inverse bremsstrahlung absorption (Fig. 3D),^105^ particularly in the presence of molecules with low ionization potentials, such as pigments. These effects are often accompanied by broadband (white light) emissions and acoustic shocks, and can result in severe cellular and tissue damage. The free electrons generated in the ionization processes can also produce strong ROS *via* interacting with water and oxygen (Fig. 3E).^106,107^

Finally, mid-IR fundamental and overtone absorption can lead to efficient sample heating (Fig. 3F), especially when the excitation wavelength matches strong vibrational absorption bands.^108–110^ The high temperature rise can significantly impact cellular function by changing lipid phase, enzyme activities, protein folding, and more. It is important to note that thermal effects occur in all the processes described in Fig. 3; the extent of heating depends on laser dose, absorption cross-sections, nonradiative relaxation efficiency, and, when ionization happens, energy transfer from hot electrons.

It is worth noting that not all photoperturbations are toxic or detrimental. Under appropriate conditions, mild optical radiation can instead promote cell proliferation and accelerate wound closure through beneficial photobiomodulation.^111–113^

### 4.2 Phototoxicity and photoperturbation in CRS microscopy

The photodamage during CRS microscopy can arise from both linear and nonlinear absorption processes.^114–116^ The dominant pathway depends on the interplay between laser parameters (peak power, wavelength, laser dosage) and the molecular composition of the sample. These processes can be classified into photochemical, photothermal, and, at extreme intensities, photomechanical damage that disrupts cellular homeostasis.

Near-infrared (NIR) laser pulses are usually used for CRS microscopy. In such cases, linear absorption is only significant when the laser average power is very high, or the excitation wavelength matches specific absorption bands of biomolecules (*e.g.*, water or lipid overtone bands, as shown in Fig. 3F).^117,118^ Nonlinear absorption, instead, is often a primary concern in CRS imaging. When the laser peak power is high, multiphoton absorption by intrinsic light absorbers,^119^ especially those with more delocalized electrons, tends to enter the triplet state through intersystem crossing, followed by the generation of ROS and RNS, as illustrated in Fig. 3A. Both type I (electron-transfer-mediated radical or peroxide formation) and type II (singlet oxygen generation) photosensitization pathways may be involved.^120^ The resulting oxidative stress leads to biomolecular damage, including DNA fragmentation, lipid peroxidation, and protein carbonylation.^121–123^ Such damage to DNA and cellular membranes can result in lethal or non-lethal functional changes,^116^ loss of cloning efficiency,^124,125^ and apoptosis.^122^

At extremely high peak powers (*e.g.*, over 10 kW), multiphoton ionization can generate plasma and free electrons, leading to chemical bond fragmentation and localized sample ablation (Fig. 3D).^126^ These free electrons can further drive an avalanche process through inverse bremsstrahlung absorption in the intense electric field of the pulsed laser (Fig. 3D).^126^ This phenomenon is often observed as continuum (white light) generation and is more likely to occur at locations containing strong absorbers with low ionization energy or free electron donors, such as metal particles, pigments, or carbon black.^127–129^ Once initiated, the avalanche process can result in severe photomechanical damage accompanied by rapid temperature increases due to hot-electron energy transfer.

In some cases, temperature rises induced by other mechanisms can further assist in such damage. For example, Raman-induced vibrational absorption is enhanced under stimulation enhancement conditions that can be comparable in magnitude to two-photon absorption.^130^ During coherent Raman resonance excitation, the SRS process deposits photon energy directly into specific molecular vibrational modes, with an efficiency of approximately 0.08%.^115^ This process creates a population of molecules in vibrationally excited states. Most of this energy is dissipated as heat, leading to local temperature increases.^33,131^ Heat accumulation, particularly when the laser remains stationary rather than scanning, can lower the damage threshold of multiphoton absorption or ionization processes.

When visible pulsed lasers are used for CRS imaging, the phototoxicity is usually significantly higher than when using the NIR laser pulses.^119,132^ Visible wavelengths can directly excite endogenous chromophores such as NADH, flavins, and porphyrins through linear absorption,^133^ introducing photosensitization effects. In addition, visible pulses are more efficiently absorbed by biomolecules through multiphoton processes, resulting in a substantially lower threshold for multiphoton ionization compared to NIR excitation at the same pulse duration and energy. To mitigate multiphoton and avalanche ionization, longer pulse durations or lower peak powers can be employed.^119^ However, these strategies do not change photodamage arising from linear absorption and ROS generation. In such cases, conventional indicators of damage, such as morphological changes or continuum generation (commonly used in tissue imaging), are insufficient for evaluating photodamage in live cells. Significant impairment of cellular function can occur well below the plasma generation threshold. More sensitive metrics for assessing phototoxicity will be discussed in later sections.

In summary, photochemical toxic effects typically occur at substantially lower laser intensities than obvious morphological damage. In CRS microscopy using NIR excitation, phototoxicity is primarily driven by multiphoton processes due to the high peak power and low average power regime. When visible pulsed lasers are employed, damage thresholds are further reduced, and linear absorption plays a more significant role. Plasma formation, free electron generation, and strong photomechanical effects represent extreme damage regimes, often identifiable through continuum generation and visible structural disruption. These events correspond to irreversible damage, whereas photochemical perturbation and toxicity occur at much lower thresholds.

### 4.3 Photodamage and signal-to-noise ratio

**Fig. 4:**
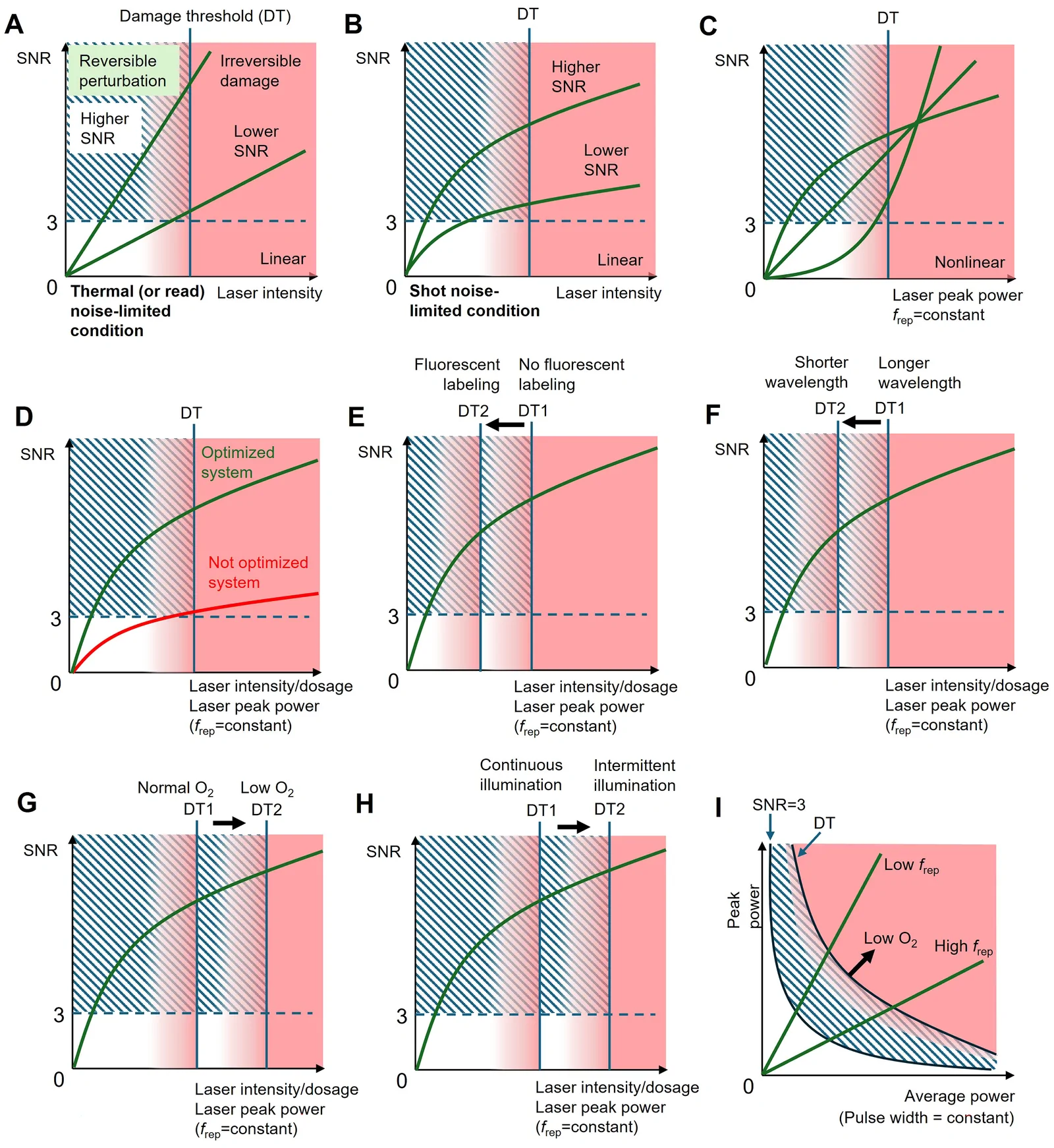
Photodamage and signal-to-noise ratio (SNR) in linear and nonlinear optical imaging. (A) Laser intensity *vs.* SNR for linear optical imaging when the dominant noise is thermal noise or detector read noise. Two lines represent molecules with different signal levels. The damage threshold (DT) separates reversible and irreversible photodamage. (B) Similar to panel A, for linear imaging processes, when the dominant noise term is shot noise. (C) Similar to panel A, but for nonlinear optical imaging. Here, SNR is plotted as a function of laser peak power while the repetition rate is held constant. Depending on the nonlinear order and laser beam, the SNR-peak-power dependence could be different. (D) Impact of system SNR optimization on the response curves. (E) Effect of fluorescent labeling on the DT. (F) Influence of excitation laser wavelength on the DT. (G) The general effect of oxygen levels on the DT. (H) Comparison of continuous *versus* intermittent illumination and their effects on the DT. (I) Combined plots of SNR, DT, and signal response as functions of laser average and peak power, with a tunable repetition rate and constant pulse width. For all panels, to detect a chemical species without inducing irreversible photodamage, the system must operate within the shaded regions. In each figure, we consider the same pixel dwell time and total imaging time.

The analysis of photoperturbation or photodamage is inseparable from the microscopy system's signal-to-noise ratio (SNR). To reliably distinguish a signal from background noise, an SNR of 3 is generally established as the LOD. While increasing excitation laser power typically improves the SNR, it also elevates photoperturbation. Once the damage threshold (DT) is surpassed, irreversible photodamage occurs. For live-cell imaging, the excitation intensity must be carefully tuned to fall within the safe operating window: above the SNR = 3 limit but below the DT (shaded areas in Fig. 4). Fig. 4A illustrates the case in which thermal noise or read noise is the dominant noise source. Under these conditions, the SNR of the imaging system depends linearly on the input laser intensity. Under shot-noise-limited conditions, the SNR generally scales with 
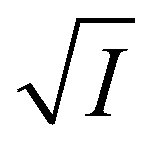
 (as shown in Fig. 4B), where *I* is the input laser intensity. In both cases, chemical species with higher intrinsic signal levels offer a wider operational range than those with lower signal levels.

In nonlinear optical imaging, the relationship between SNR and peak power depends on the specific laser beam under consideration and the order of the nonlinear optical process. For example, under shot-noise-limited conditions, the signal of SRS is proportional to *I*_p_*I*_s_. When the Stokes beam is modulated and the pump beam is detected, the shot noise is proportional to 
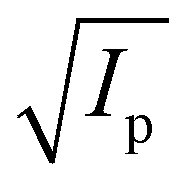
. Therefore, the SNR is proportional to 
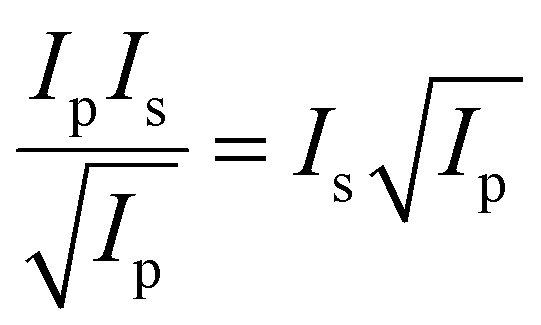
. For CARS, the signal is proportional to *I*_p_^2^*I*_s_, while the noise is proportional to 
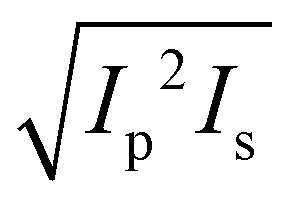
, resulting in an SNR that is proportional to 
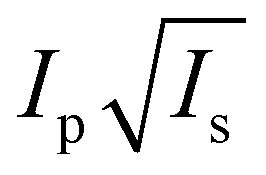
. For three-photon excitation fluorescence, the SNR is proportional to 
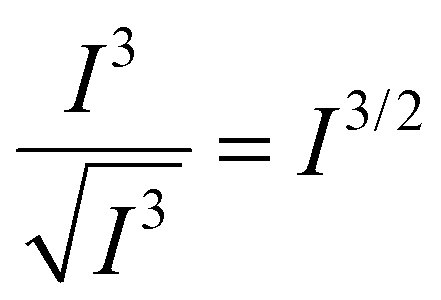
. These scaling relationships represent ideal shot-noise-limited conditions and do not account for background contributions, detector noise, or other electronic noise sources. Fig. 4C summarizes these relationships for nonlinear optical modalities. We use the 
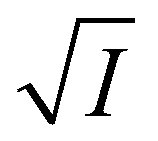
 relationships as an example for the following figure panels.

Furthermore, system optimization may be critically important, especially for CRS microscopy. Here, “optimization” refers to fine-tuning the optical system to achieve the highest possible SNR for a given input laser power. In fluorescence microscopy, for example, optimization may involve adjusting the pinhole position to maximize signal transmission, using lenses with appropriate optical coatings, and optimizing other optical parameters. In CRS microscopy, optimization examples include achieving optimal spatial and temporal overlap between the pump and Stokes pulses, matching the pulse chirping parameters, and optimizing beam divergence, beam size, and beam quality, among other factors. Each imaging system typically has its own benchmark SNR for a standard sample under a fixed excitation condition. To facilitate comparisons across different CRS systems, generic performance metrics, such as LOD of a standard sample, can also be used as system-independent benchmarks. As shown in Fig. 4D, an optimized system can achieve the same SNR at a lower excitation power, thereby reducing photoperturbation. In contrast, an unoptimized system requires substantially higher laser power to reach comparable SNR, increasing phototoxicity. Compared to CRS, fluorescence microscopy typically achieves high SNR at relatively low excitation powers and is therefore more tolerant to misalignment. Because CRS imaging usually has weak signals and often operates at high laser powers, its operating curve could be inherently closer to the SNR = 3 and DT boundary. When the system is unoptimized, the operating power range may fall entirely outside the safe region for longitudinal live-cell imaging.

Multiple factors can shift the DT under otherwise similar imaging conditions. For example, fluorescent labeling often increases phototoxicity by assisting ROS generation, thereby lowering the DT (Fig. 4E).^3,92^ Shorter excitation wavelengths, particularly in the blue or UV range, can also significantly reduce the DT (Fig. 4F). Conversely, reduced O_2_ levels tend to increase the DT by reducing ROS generation (Fig. 4G).^92,134^ However, the lack of oxygen might also negatively impact cellular functions. Intermittent illumination can elevate the DT compared to continuous exposure, as it allows ROS to dissipate and be scavenged more effectively (Fig. 4H).^135^

Finally, because CRS is a nonlinear optical process, the laser repetition rate provides an additional degree of freedom. When tunable (*e.g.*, *via* the pulse-picking method^136^), it modifies both SNR and DT constraints, as illustrated in Fig. 4I. Lower repetition rates can improve per-pulse signal generation but often reduce the allowable average power before reaching the DT. In practice, the optimal balance among laser power, SNR, and DT is highly dependent on the specific sample and imaging parameters.

### 4.4 Signal-to-perturbation ratio

#### 4.4.1 CRS signals

The signal of CRS at each pixel is the accumulation of photons involved in the Raman transition process over the pixel dwell time. The signal of CARS per pixel satisfies1
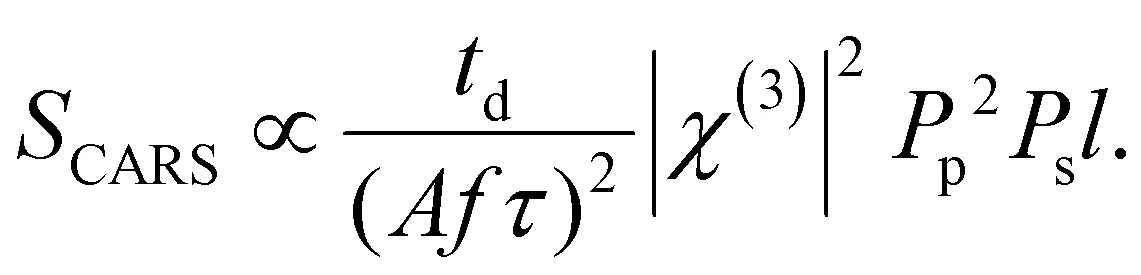


Here, *t*_d_ is the pixel dwell time, *f* is the repetition rate, *τ* is the pulse width, *A* is the laser focus beam size, *l* is the interaction length, and *P*_p_ and *P*_s_ are the average laser power values, respectively. The quantity *S*_CARS_ is the average signal energy since it is the average signal power multiplied by the pixel dwell time. In this expression, the pump and Stokes laser powers are assumed to remain constant over time. We are also assuming the pump and Stokes beams have the same values for *A*, *f*, *τ*, and *l*. The term *χ*^(3)^ is the macroscopic third-order optical susceptibility of the analyte, which depends on molecular concentration. It is proportional to the ensemble sum of orientation-weighted third-order molecular hyperpolarizabilities *γ*(*ω*) of the target molecules^137,138^

2 *χ*^(3)^∝*n*〈*γ*(*ω*)〉,

where *n* is the number density of molecules under detection. The *S*_CARS_ can then be written as:3
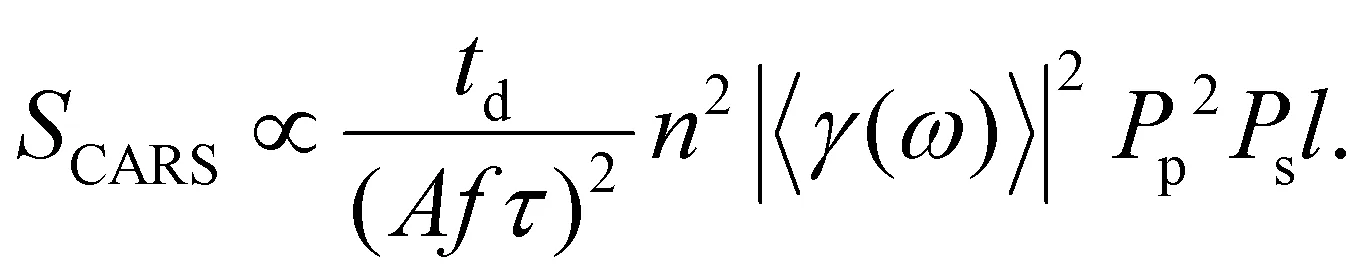


For SRS, the overall signal per pixel satisfies:4
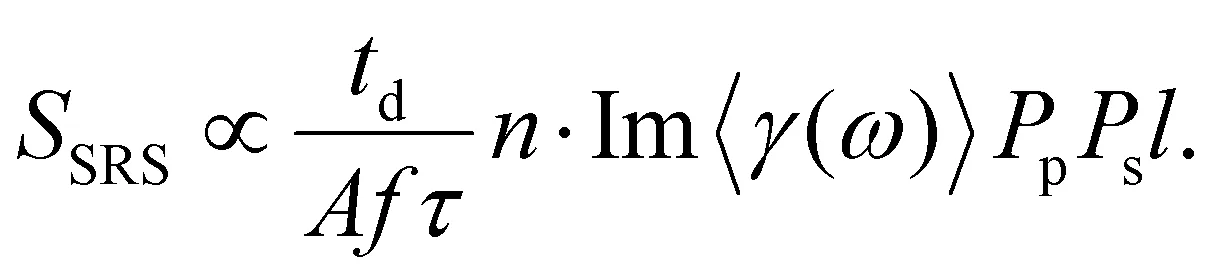


For both CARS and SRS, if the input average powers are constant, shorter pulses, lower repetition rate, smaller pixel size, and longer pixel dwell time give stronger signals.

#### 4.4.2 Optical perturbation at each pixel using pulsed lasers

Considering only the signal level or SNR is insufficient for longitudinal live-cell imaging. As illustrated in Fig. 4, the imaging system must operate within the safe window below the DT and above the LOD of SNR = 3 (shaded regions). System optimization in SNR can generally shift the SNR curves toward lower excitation powers, enabling improved SNR at the same laser input power.

The DT is influenced by multiple factors, including laser average power, wavelength, repetition rate, pulse duration, pixel dwell time, focal spot size, scanning area, and total exposure time. To evaluate photoperturbation, we first consider the effect on individual image pixels. As discussed earlier, when NIR lasers are used for excitation, nonlinear absorption becomes the dominant concern compared with linear absorption. Under these conditions, the peak power primarily determines the efficiency of multiphoton absorption and, in extreme cases, ionization processes, whereas the average power governs the cumulative dose deposited through the multiphoton interactions induced by successive laser pulses.

Taking two-photon absorption (TPA) as an example,^139,140^ the excitation rate per molecule can be written as:

5 *R*_TPA_ = *σ*_2_*Φ*^2^

where *σ*_2_ is the two-photon absorption cross-section, and *Φ* is the photon flux, which satisfies6
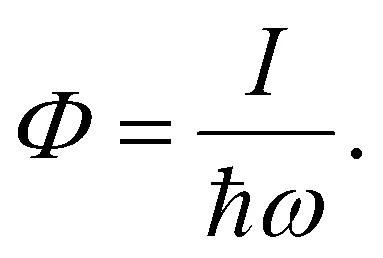


The absorption power per molecule then becomes

7 *P*_molecule_ = 2*ħωσ*_2_*Φ*^2^

If the laser is focused to the size of *A* and an interaction length of *l*, and the number density of the molecule is *n*, we can obtain the total TPA power8
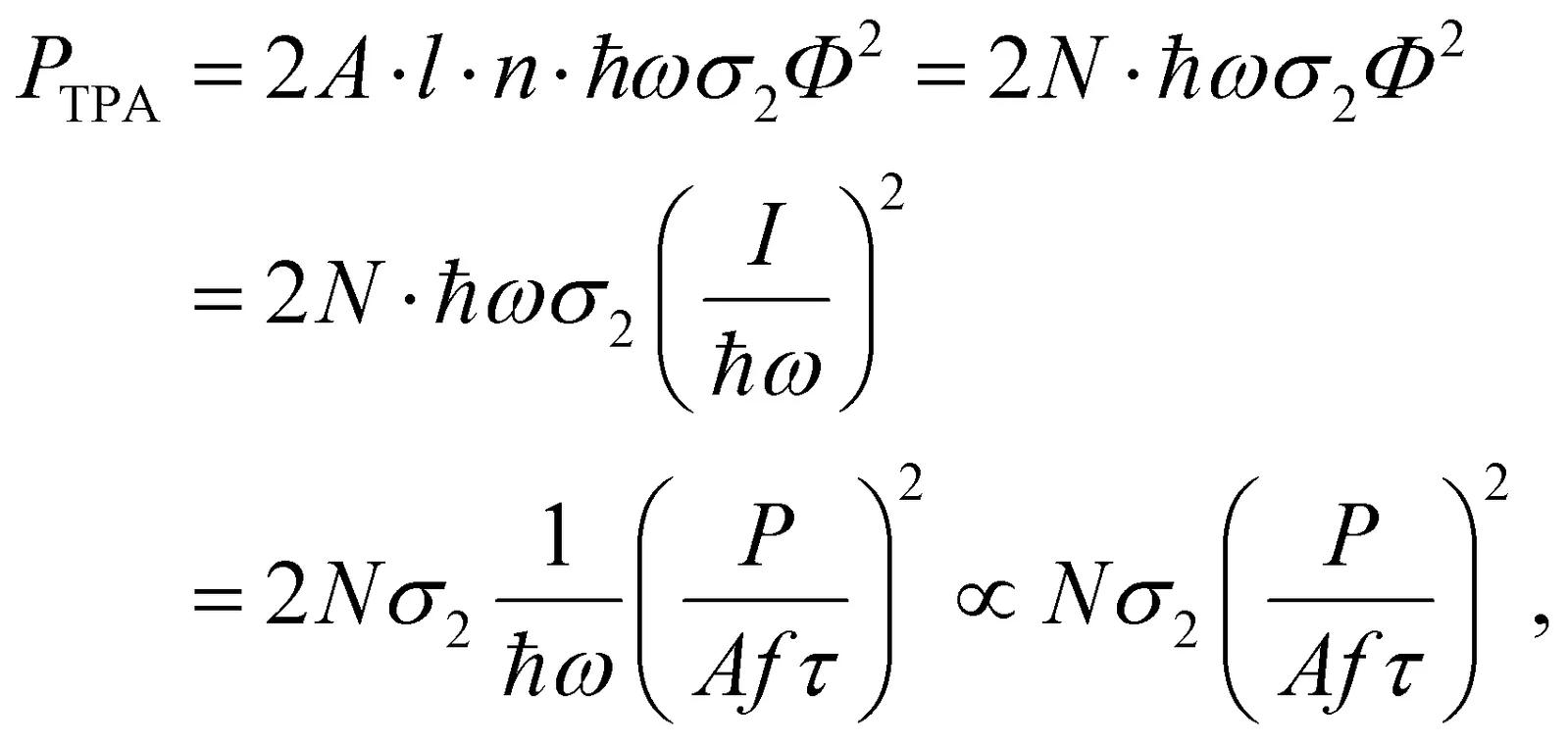
where *N* is the total number of molecules contributing to the TPA process.

A generalized expression for optical perturbation per pulse that summarizes a class of pulsed-laser-induced mechanisms illustrated in Fig. 3 (excluding linear absorption induced by CW lasers and avalanche ionization processes) can be written as:9
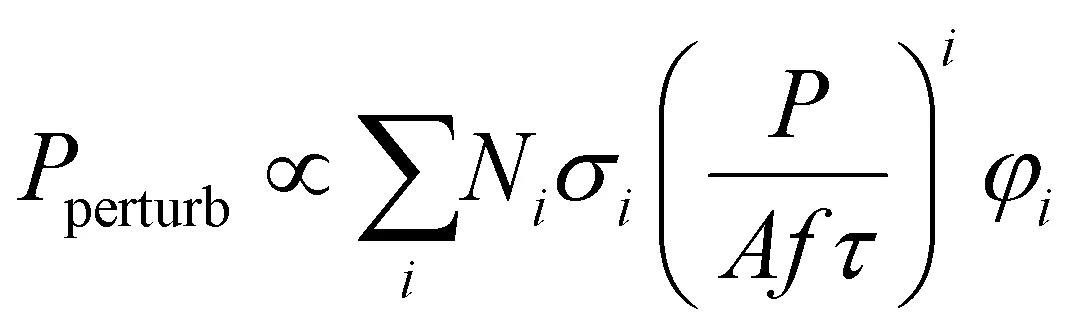


Here, *P* is the average power of the laser pulse train, *f* is the repetition rate, *τ* is the pulse width, and *A* is the laser focus beam size. *P*/(*Afτ*) represents the peak power density of the laser pulses. In addition, *i* denotes *i*-photon processes, respectively; *σ*_*i*_ is the corresponding multiphoton absorption cross-section (frequency-dependent, unit: cm^4^ s per molecule for two-photon processes), *N*_*i*_ is the total number of molecules that satisfy *N*_i_ = *A*·*l*·*n*_*i*_, where *n*_*i*_ is the number density of molecules related to the *i*^th^ multiphoton process.

The coefficient *φ*_*i*_ represents the efficiency of the downstream photoperturbation process associated with the *i*^th^-order absorption event. For example, when *φ*_*i*_ corresponds to triplet-state conversion, the corresponding term in eqn (9) represents the cumulative contribution of nonlinear absorption processes to triplet-state-associated photoperturbation (Fig. 3A and B). If *φ*_*i*_ describes direct molecular breakdown induced by multiphoton absorption (Fig. 3C), the corresponding term in eqn (9) quantifies such photodamage-related perturbation. When *φ*_*i*_ represents nonradiative decay, the corresponding term in eqn (9) corresponds to the absorbed power contributing to photothermal effects.

The excitation wavelength primarily influences *σ*_*i*_ and the accessible perturbation orders *i*. When a multiphoton process coincides with a resonant multiphoton absorption transition, the corresponding *σ* value can increase substantially. These cross-sections are also strongly influenced by the electronic structures of the molecules within the laser focal volume.

Let's assume the laser perturbation from the pulsed laser is linearly scaled with the number of laser pulses. At each pixel, the laser interaction time *T* = *t*_d_*fτ*.

Therefore, the total energy contributed to photoperturbation is10
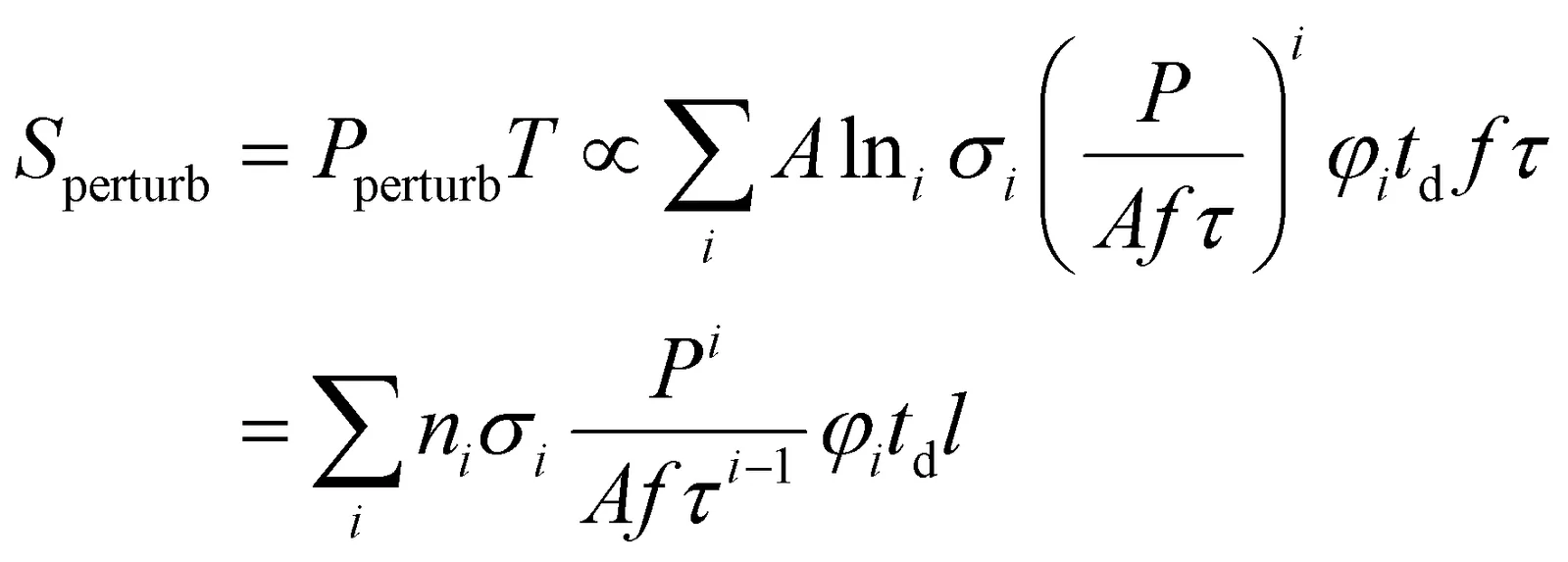


If we consider the different processes described in Fig. 3, we can obtain11
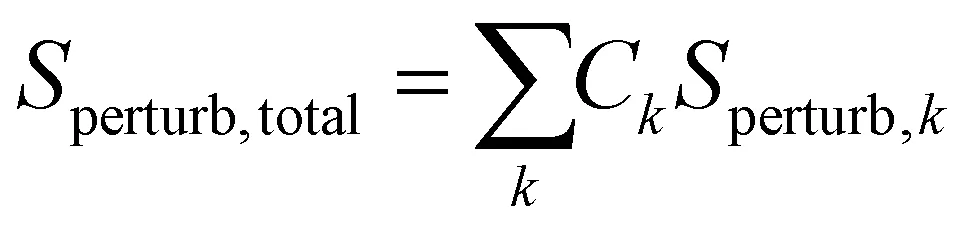
where *C*_*k*_ is the factor that weighs the perturbation from each process *k*. In general, for multiphoton imaging of live cells*C*_ionization+avalanche_ ≫ *C*_triplet state ROS_ > *C*_photothermal_

#### 4.4.3 An example of two-photon absorption-associated triplet-state perturbation

Considering only the perturbation that contributes by two-photon absorption-associated triplet-state conversion, eqn (8) becomes,12
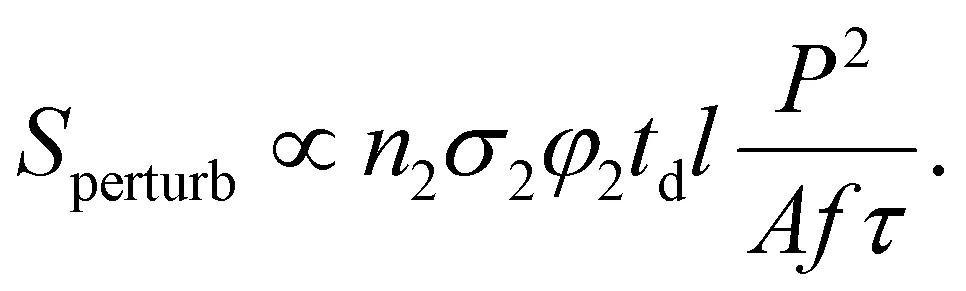


For CRS microscopy, if both the pump and Stokes pulses are involved, both degenerate and nondegenerate two-photon absorption can contribute. Therefore, *S*_perturb_ becomes13
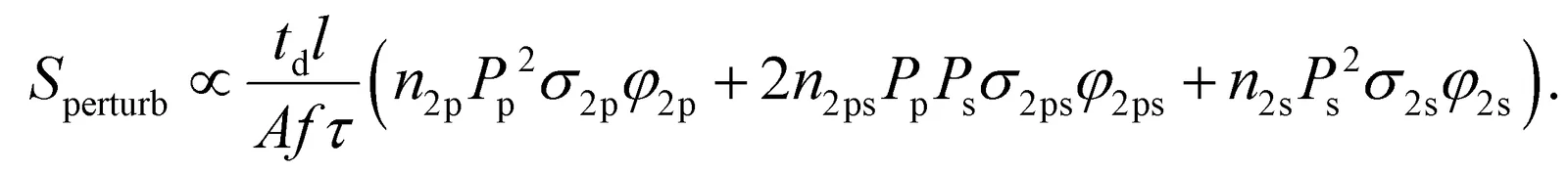


Practically, the laser power needs to be chosen to ensure that this *S*_perturb_ per pixel is below the DT value, which is mostly empirical and sample-dependent. It is highly influenced by the laser settings (*P*, *A*, *f*, *τ*), imaging configurations (*t*_d_), molecular concentration (*N*), and molecular properties (*σ* and *φ*). Here, we are not considering cell size and organelle-specific photoperturbation, which will be discussed in a later section.

#### 4.4.4 Signal-to-perturbation ratio

We define signal-to-perturbation ratio (SPR) as the ratio of *S*/*S*_perturb_. This ratio quantifies the signal level relative to the photoperturbation impact for the given modality. A higher SPR indicates that relatively higher signals can be achieved with the same photoperturbation to the sample.

For simplicity, we only consider the previously discussed two-photon absorption-induced triplet-state conversion process for photoperturbation.14
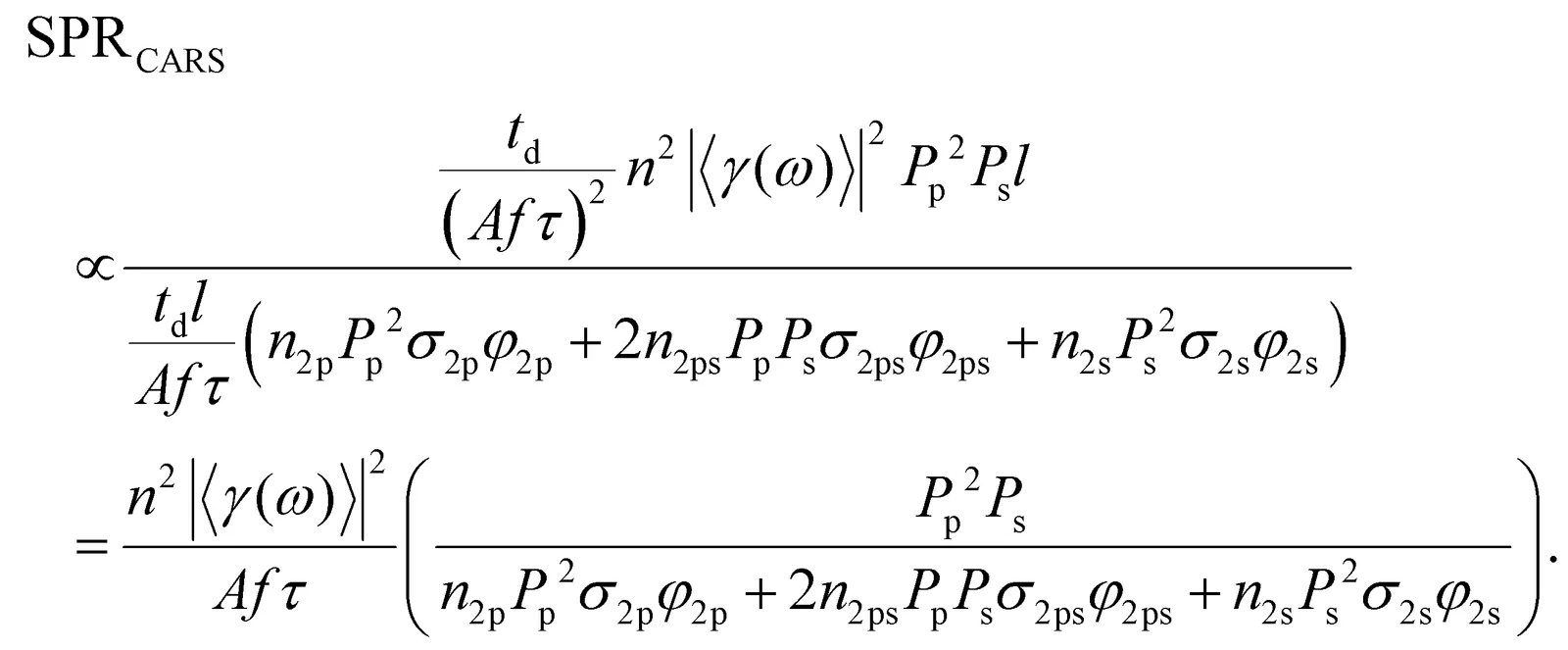


Let's further simplify this expression by assuming *φ*_2p_ ≈ *φ*_2ps_ ≈ *φ*_2s_ ≈ *φ*_2_, *σ*_2p_ ≈ *σ*_2ps_ ≈ *σ*_2s_ ≈ *σ*_2_, *n*_2p_ ≈ *n*_2ps_ ≈ *n*_2s_ ≈ *n*_2_, the results become:15
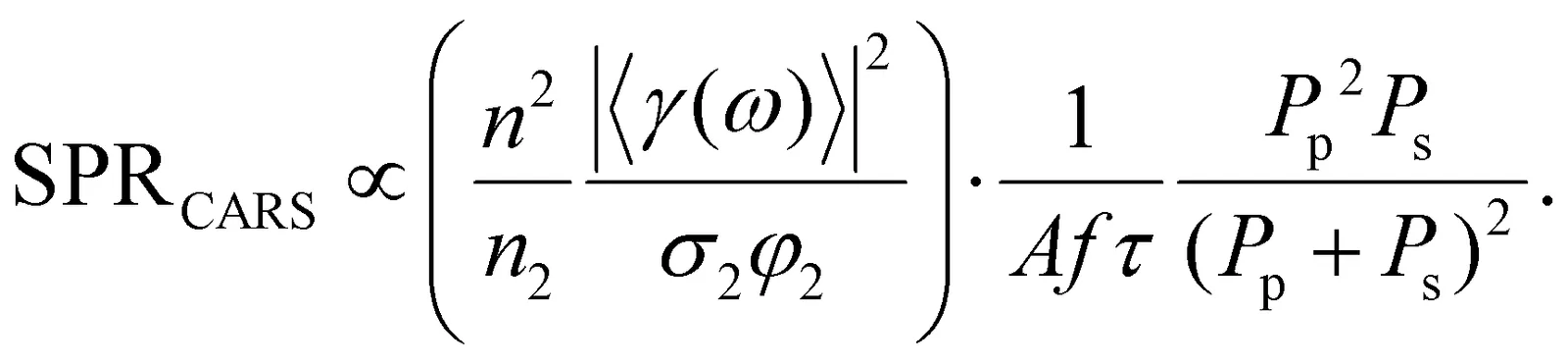


This expression shows how the molecular properties and laser parameters influence the SPR for CARS. A higher number of molecules with desired Raman responses (*n*), higher Raman transition responses <*γ*(*ω*)> at resonance, a lower number of molecules contributing to the two-photon absorption process (*n*_2_), a lower TPA cross-section, and a lower triplet-state conversion rate (*φ*_2_) increases the SPR; lower repetition rate (*f*), shorter pulse width (*τ*), and higher pump laser power (*P*_p_) give higher SPR. The laser power factor will be discussed in the next section. Practically, these considerations indicate that to improve SPR of CARS if two-photon absorption related perturbation dominates, one can reduce the contribution of two-photon absorption, lower the intersystem crossing rate, and use lower repetition rates, shorter pulse widths, and smaller focal spots.

It is worth noting that the SPR value does not determine the DT value. It reflects the relative efficiency of generating optical signals *versus* inducing photoperturbation. Theoretically, SPR should be unitless. However, due to the difference in units between *γ*(*ω*) and *σ*_2_, the right-hand side of eqn (13) is not strictly unitless. Nevertheless, this does not affect comparative analysis when only relative changes in SPR (*e.g.*, increases or decreases) are considered, since the unit discrepancy is accounted for by the proportional relationship.

Similarly, we can obtain16
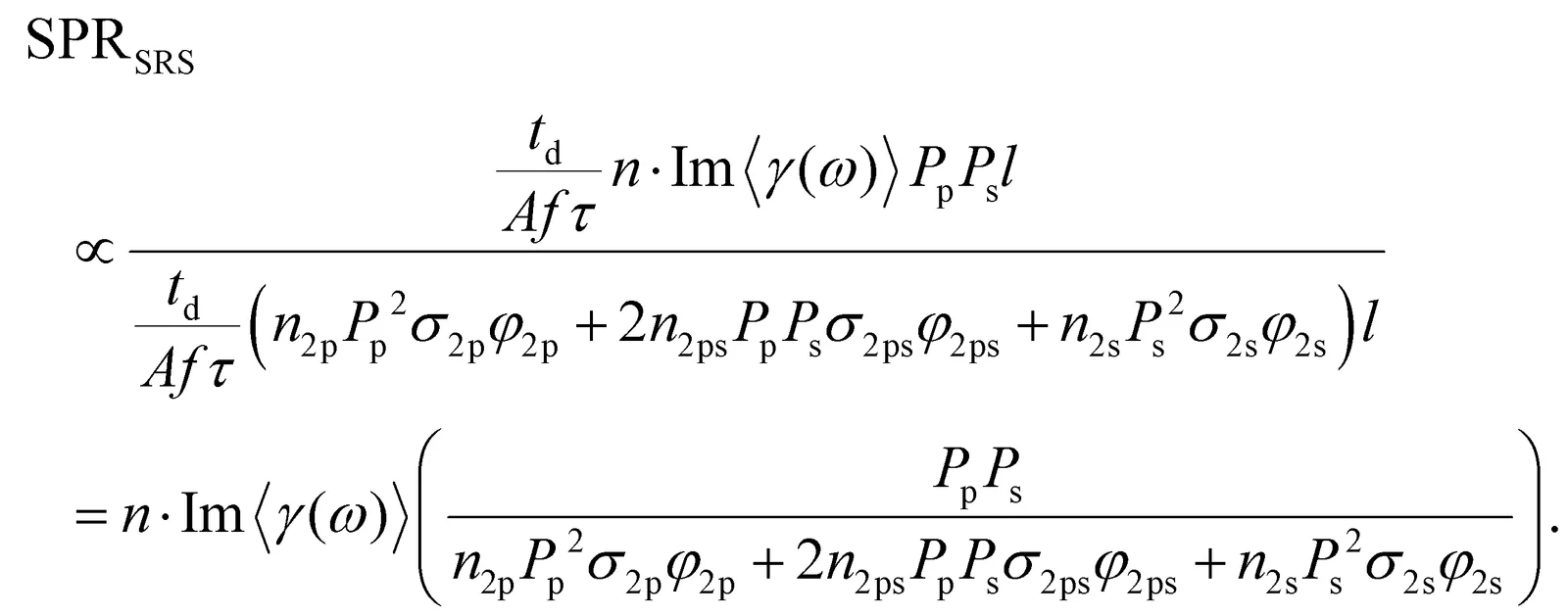


Assuming *φ*_2p_ ≈ *φ*_2ps_ ≈ *φ*_2s_ ≈ *φ*_2_, *σ*_2p_ ≈ *σ*_2ps_ ≈ *σ*_2s_ ≈ *σ*_2_, *N*_2p_ ≈ *N*_2ps_ ≈ *N*_2s_ ≈ *N*_2_, the result becomes:17
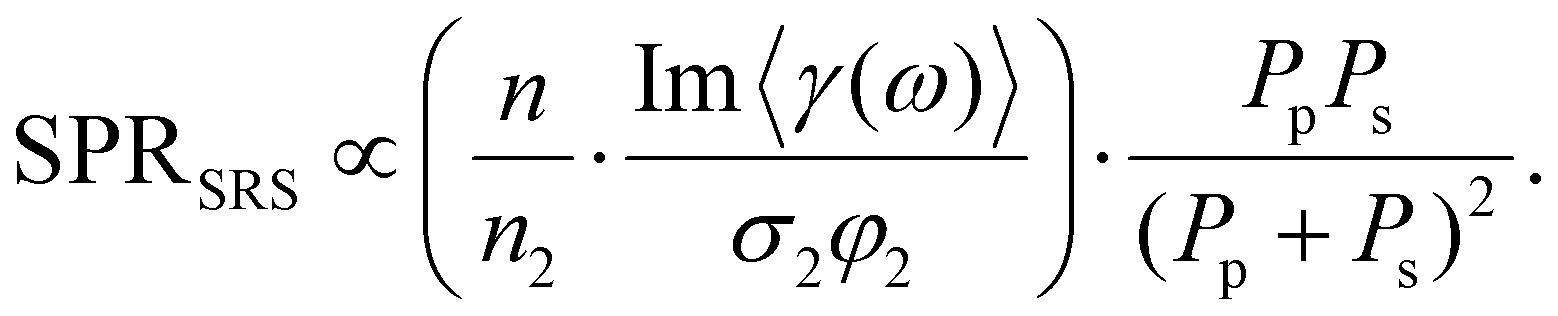


This result indicates that, for SRS, the SPR (considering TPA only) is independent of the laser repetition rate and pulse width. The molecular response factors are similar to those in CARS SPR, except that the dependence on *n* is linear rather than quadratic, as for CARS. The power factor is symmetric for *P*_p_ and *P*_s_, and will be discussed in the next section.

#### 4.4.5 Laser configurations

From eqn (15) and (17), we found that the laser configuration difference can impact *S*_perturb_ and SPR depending on *P*, *A*, *f*, *τ* values, and their combinations. To simplify this analysis, we first focus on the following factor in eqn (10):
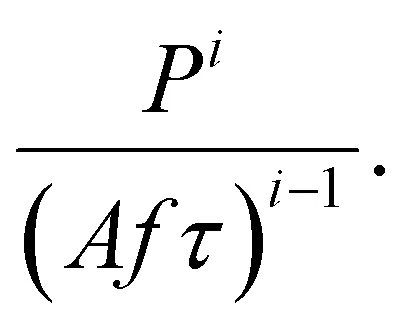


Direct multiphoton perturbation can be expressed as a simple linear combination of terms involving this factor. By setting *i* = 1, 2, 3, 4, and 5, and deriving the corresponding *S*_perturb_ and SPR expressions for each case, the factors containing only *Afτ* are summarized in Table 3. The value *ξ* = 1/*Afτ* is termed here the flux scaling factor of the focused pulsed laser. A higher value of *ξ* indicates that a higher photon or energy flux is associated.

**Table 3:** The dependence of photoperturbation and SPR on the flux scaling factor

	*S*_perturb_ ∝ *ξ*^*i*−1^		SPR_CARS_ ∝ *ξ*^3−*i*^		SPR_SRS_ ∝ *ξ*^2−*i*^	
*i* = 1	1	*ξ* ^0^	(*Afτ*)^−2^	*ξ* ^2^	(*Afτ*)^−1^	*ξ* ^1^
*i* = 2	1/*Afτ*	*ξ* ^1^	(*Afτ*)^−1^	*ξ* ^1^	1	*ξ* ^0^
*i* = 3	1/(*Afτ*)^2^	*ξ* ^2^	1	*ξ* ^0^	*Afτ*	*ξ* ^−1^
*i* = 4	1/(*Afτ*)^3^	*ξ* ^3^	*Afτ*	*ξ* ^−1^	(*Afτ*)^2^	*ξ* ^−2^
*i* = 5	1/(*Afτ*)^4^	*ξ* ^4^	(*Afτ*)^2^	*ξ* ^−2^	(*Afτ*)^3^	*ξ* ^−3^

This table shows, under the same average power condition, how the laser beam size *A*, repetition rate *f*, and pulse width *τ* influence the optical signal and perturbations differently. The same flux scaling factor *ξ* resulting from different *A*, *f*, *τ* combinations should theoretically give the same perturbation. For the nonlinear effect associated with optical perturbations, it scales with *ξ*^*i*−1^. The CARS signal is proportional to *ξ*^2^, while the SRS signal is proportional to *ξ*. Therefore, the CARS SPR scales with *ξ*^3−*i*^ for the *i*^th^ process. The SRS signal is proportional to *ξ*; therefore, the SRS SPR scales with *ξ*^2−*i*^ for the *i*^th^ process.

These values indicate that a higher photon or energy flux gives a higher multiphoton optical perturbation. If higher-order multiphoton processes dominate the photodamage, reducing flux would give better SPR values. This regime is particularly important when multiphoton ionization processes are involved. When linear absorption is dominant in photoperturbation, a higher flux is preferred because it more effectively generates CARS or SRS signals.

Because the repetition rate *f* and pulse width τ are usually fixed for a given excitation laser source, the flux scaling factor *ξ* is therefore primarily determined by spot size A, which is largely determined by the focusing numerical aperture (NA) of the objective lens. Since the laser spot diameter is inversely proportional to NA, the flux scaling factor therefore scales approximately as *ξ* ∝ (NA)^2^. A higher NA thus results in a higher flux scaling factor and, consequently, a higher excitation flux. The contribution of this increased flux to SPR depends on the dominant mechanism responsible for laser-induced perturbation.

Assuming *φ*_*i*p_ ≈ *φ*_*i*ps_ ≈ *φ*_*i*s_ ≈ *φ*_*i*_, *σ*_*i*p_ ≈ *σ*_*i*ps_ ≈ *σ*_*i*s_ ≈ *σ*_*i*_, *N*_*i*p_ ≈ *N*_*i*ps_ ≈ *N*_*i*s_ ≈ *N*_*i*_, the power factor for CARS for the *i*^th^ process becomes18
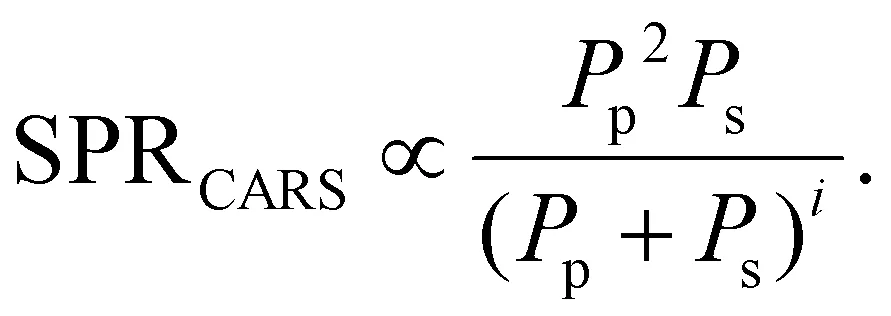


And for SRS, it becomes19
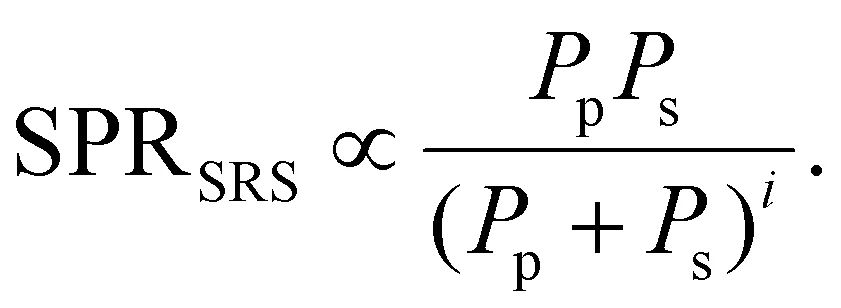


**Fig. 5:**
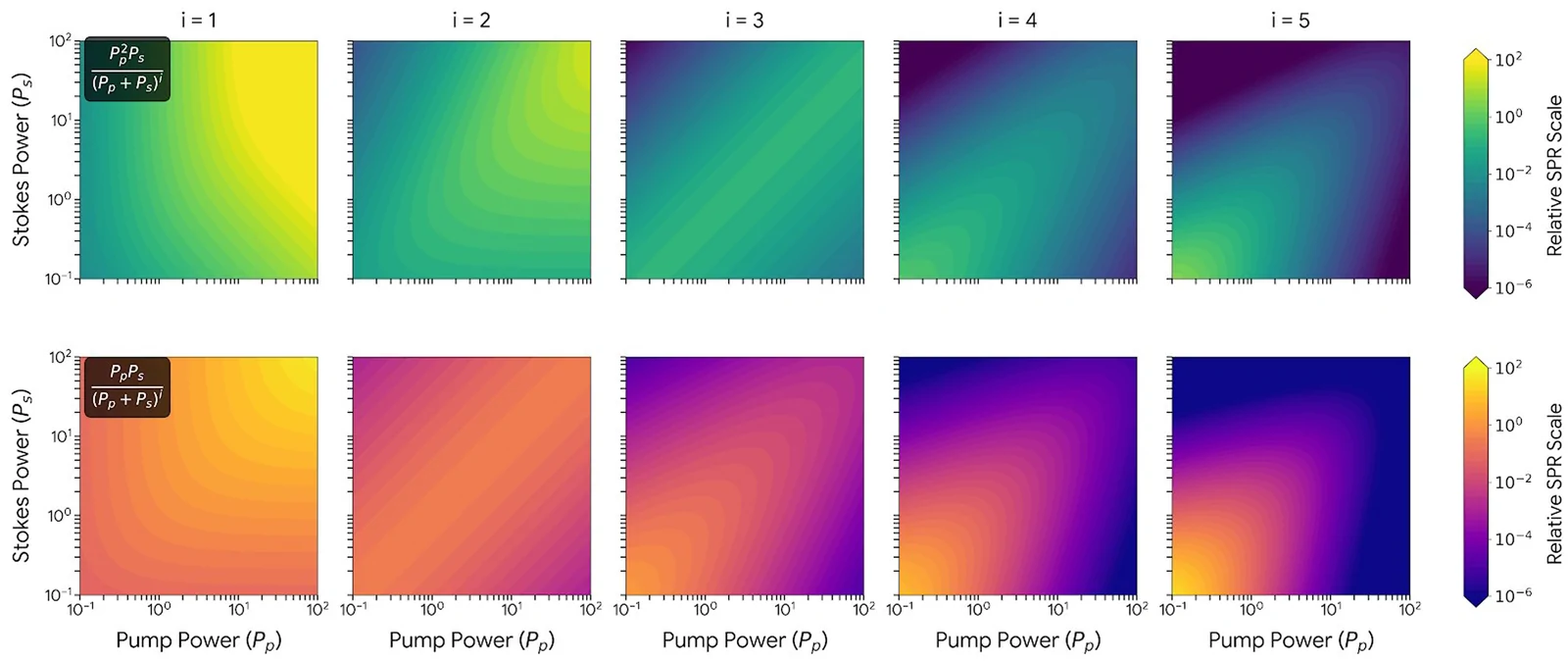
Contour plots of the signal-to-perturbation ratio (SPR). The SPR values as a function of *P*_s_ and *P*_p_ at different values of *i* (perturbation optical order) for CARS (top) and SRS (bottom). The values of *i* = 1, 2, 3, 4, 5 are selected. The figures are plotted on log scales for each case.

Contour plots of the SPR as functions of *P*_p_ and *P*_s_ are shown in Fig. 5. The results indicate that if the total input power *P*_p_ + *P*_s_ is constant, higher *P*_p_ gives better CARS SPR due to the square of *P*_p_ in the numerator. For SRS, since the expression is symmetric for *P*_p_ and *P*_s_, the plots also show symmetric configurations. Furthermore, for CARS, when *i* < 3, higher average power gives better SPR, while when *i* = 3, the SPR is not power dependent, and when *i* > 3, lower average power gives better SPR. A similar trend is seen for SRS, but the cutoff nonlinear optical order is *i* = 2. When detector saturation limits and the wavelength dependence of the Raman cross section (*σ*) are neglected, the optimal pump-to-Stokes power ratio (*P*_p_ : *P*_s_) is 2 : 1 for CARS and 1 : 1 for SRS.

#### 4.4.6 Linear absorption using CW lasers

This SPR framework can be readily adapted for linear optical imaging using CW lasers. In such cases, peak power and laser repetition rate no longer exist. The fluorescence emission signal under linear excitation can be expressed as

20 *S*_F_ ∝ *PAlt*_d_*nσϕ*,

where *ϕ* is the quantum yield of fluorescence emission.

The photoperturbation related to triplet state conversion can be expressed as

21 *S*_perturb_ ∝ *PAlt*_d_*nσφ*,

where *φ* is the triplet state conversion efficiency.

Therefore,22
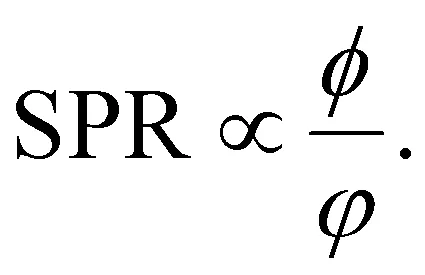


This indicates that the SPR is mostly determined by the ratio of the quantum yield of the fluorophore and the triplet state conversion efficiency if triplet-state-mediated ROS is the dominant toxicity factor.

#### 4.4.7 Optical perturbation for live cells

We have so far only considered photoperturbation on a per-pixel basis. In practice, cell size should also be considered during illumination, as larger cells can receive a greater total laser dosage. On the other hand, larger cells may exhibit higher tolerance to photoperturbation. This effect can be incorporated into SPR by introducing an additional factor:23
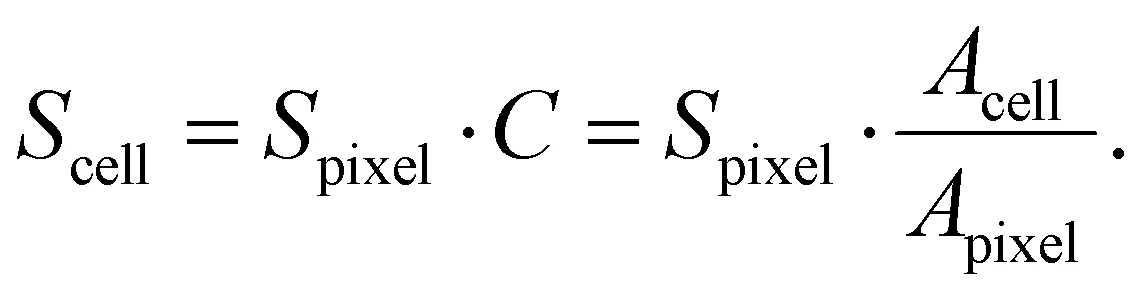


Here, *C* is the number of pixels covered by the cell. *A*_cell_ stands for the cell size, while *A*_pixel_ indicates pixel size. This expression is only theoretical and simplified. In reality, different organelles exhibit diverse responses to photoperturbation due to their distinct chemical compositions and biological functions. For example, cellular compartments containing stronger optical absorbers, such as flavins, NADH, or pigments, can experience substantially higher photoperturbation than other regions. In addition, the same optical perturbation may produce different biological consequences depending on the organelle in which it is generated. For instance, ROS generated in mitochondria, in comparison to LDs, may cause more severe short-term cellular responses because of impaired ATP production.^141^

Furthermore, in comparison with linear absorption-related photoperturbation, multiphoton-induced photoperturbation at low excitation levels is much more spatially confined with much shorter axial interaction length.^128^ The actual photoperturbation values and DT are highly case-dependent and can only be evaluated empirically using the metrics discussed in the following section.

Overall, SPR provides a theoretical framework for evaluating how laser parameters and molecular properties jointly influence the signal-phototoxicity trade-off. The optimal imaging parameters depend on the dominant perturbation mechanism: higher excitation flux can improve SPR when linear processes dominate, whereas reducing excitation flux can be advantageous when higher-order nonlinear processes contribute substantially to photoperturbation.

### 4.5 Metrics to evaluate phototoxicity in optical microscopy

As discussed before, photoperturbation to biological systems depends on many factors, including laser configurations, sample properties, and interaction time. There is no universal DT that separates live and dead cells after imaging. The best way to evaluate phototoxicity and photoperturbation is time-lapse imaging, ideally longitudinal over a long enough period for evaluation with sufficient chemical or morphological feature changes. Although many research groups have reported the evaluation of photodamage using various laser configurations, the definition of ‘damage’ is quite different.

#### 4.5.1 Morphological indicators of photoperturbations

**Fig. 6:**
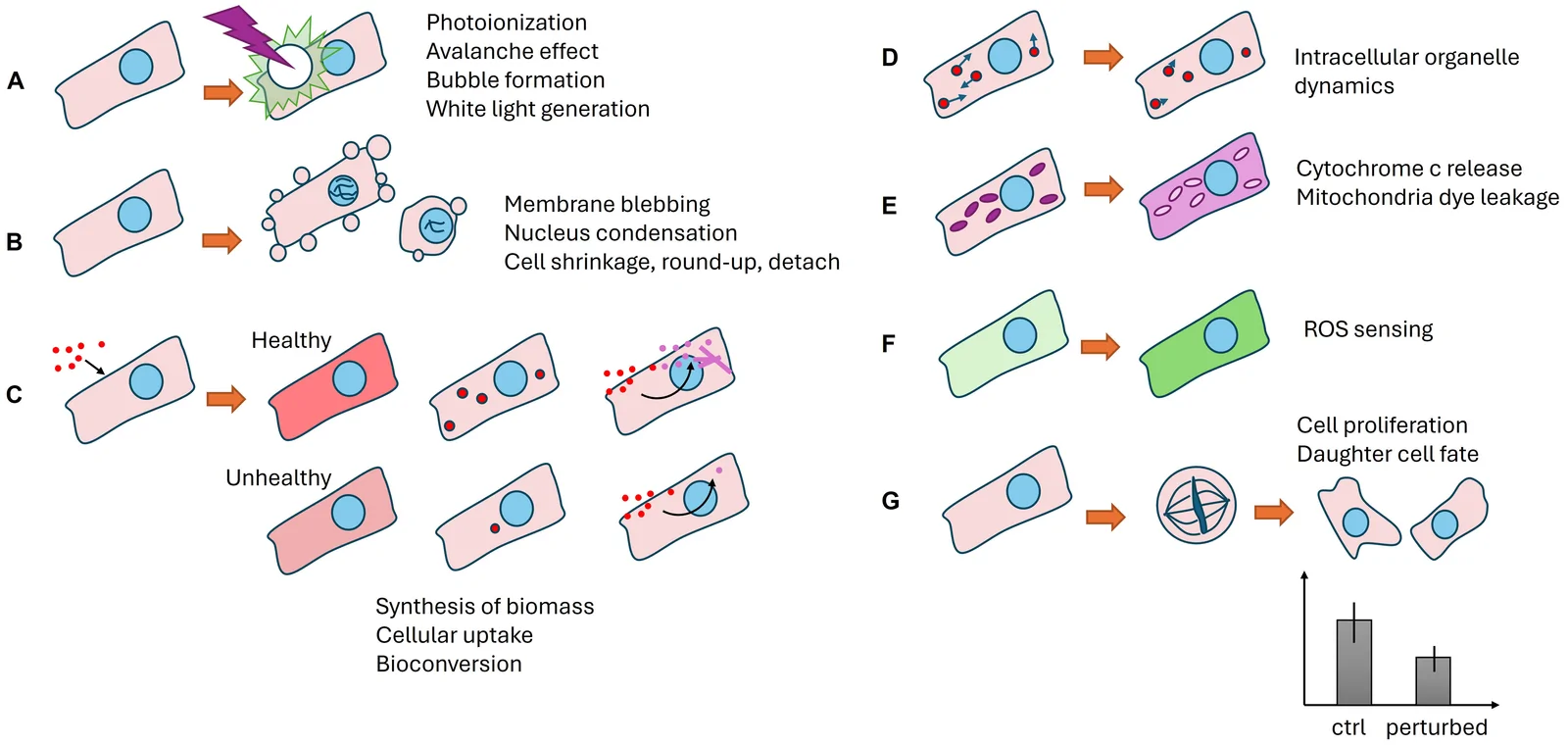
Metrics for evaluating phototoxicity and photoperturbation in live cells. (A) Photoionization and avalanche effects in combination typically lead to bubble formation and white-light generation. This is often accompanied by photomechanical forces at the burning spot. (B) Membrane blebbing, nuclear condensation, cell shrinkage, cell rounding, and detachment are hallmarks of strong photochemical perturbation leading to cell death. (C) Biomass conversion differs between healthy and unhealthy cells. (D) Altered intracellular organelle dynamics can serve as indicators of photoperturbation. (E) Cytochrome c release indicates early-stage apoptosis, whereas mitochondrial dye leakage reflects mitochondrial damage and loss of membrane potential. (F) ROS-sensitive dyes or genetically encoded sensors can detect ROS generated by the imaging light source. (G) Longitudinal monitoring of cellular functions, including mitotic progression, daughter cell fate, and overall proliferation rate, can provide important metrics for assessing phototoxicity and photoperturbation.

Morphological changes are often the most straightforward indicators of photodamage. However, they typically only reveal damages that produce pronounced structural alterations, either under severe conditions or at late stages. In conventional optical imaging, laser energy and peak power are generally kept below the ionization threshold of biomolecules. Nevertheless, in the presence of strongly absorbing materials or species with low ionization energy, significant local heating or multiphoton ionization can occur. Once free electrons are generated, they can be accelerated by tightly focused pulsed lasers, triggering avalanche ionization. Under these conditions, pronounced morphological disruptions such as bubble formation, sample deformation, plasma generation, and white-light emission may occur (Fig. 6A). For example, cumulative exposure doses of approximately 1111.6 J cm^−2^ have been reported as a “near-burning” regime under tightly focused NIR picosecond pulsed irradiation.^123^

Below such catastrophic thresholds, phototoxicity-induced cell death typically manifests as membrane blebbing, nuclear condensation, cell rounding, boundary retraction, shrinkage, and detachment (Fig. 6B). These features are commonly associated with apoptosis or necrosis, arising either from direct membrane damage *via* ROS or low-density plasma, or indirectly through disruption of essential cellular functions that ultimately compromise cytoskeletal and membrane integrity.^123,142,143^ Organelle morphology, when detectable, can serve as a highly sensitive indicator of phototoxicity. For example, mitochondrial swelling is a common hallmark of mitochondrial damage.^144^ Extensive mitochondrial damage in live cells often indicates severe phototoxicity and can ultimately lead to cell death.^121,145^ These morphological changes generally occur over minutes to hours, after which cells may undergo necrotic disintegration or remain structurally intact while nonviable.

It is important to note that although membrane blebbing has commonly been used as a morphological hallmark of apoptosis, it is neither specific to nor necessary for apoptosis.^146–149^ For example, transient blebbing can occur in healthy cells during processes such as adhesion and spreading on a substrate, without being associated with cell death.^150,151^ Conversely, apoptosis can occur without prominent membrane blebbing. Thus, membrane blebbing should be interpreted together with other morphological or functional indicators when assessing phototoxicity and cell death.

#### 4.5.2 Changes in chemical processes as readouts

Changes in cellular chemical processes could also be used as a sensitive readout for photo-perturbation. These chemical changes span from proteins to metabolites to ions, and can reflect photoperturbation from mild to strong conditions, and could be very sensitive to the initial response to perturbation.

For example, new biomass synthesis can be tracked by monitoring the incorporation of deuterated amino acids or D_2_O.^152–154^ Quantifying these metabolic incorporation rates provides a sensitive metric for determining whether imaging perturbs the cell's native biosynthetic activity.^153^ Similarly, longitudinal CRS studies have used LD biosynthesis as an indicator of laser perturbation after continuous imaging.^155^ Increased LD formation is considered to be a marker of strong alteration of cellular functions by the imaging process. In addition, increased endogenous two-photon excited fluorescence (TPEF) can serve as a real-time intrinsic marker of photodamage during label-free multiphoton imaging.^103,121^ These alterations in biomass synthesis generally require substantial biomarker accumulation and typically occur on timescales ranging from minutes to hours.

Significantly damaged cells can also be evaluated using conventional assays such as trypan blue^156^ uptake or MTT conversion (Fig. 6C),^157^ which could give the readout at a similar or faster time scale. However, these approaches are generally not applicable at the intermediate time points during longitudinal live-cell imaging.

Longitudinal imaging is uniquely suitable for tracking dynamic cellular processes, which are often more sensitive to early-stage phototoxicity than morphological features. For example, LD motility features such as displacement, velocity, and travel distance provide a label-free indicator of metabolic health (Fig. 6D).^136,158,159^ For cells that do not contain abundant LDs, other cellular organelles, such as mitochondria and lysosomes, can be used to assess cellular dynamics. These organelles can be visualized using label-free imaging techniques, including phase-contrast, dark-field, and quantitative phase imaging.^160–162^ These dynamic signatures can be quantitatively analyzed to detect changes induced by external perturbations, including laser-induced stress. Cellular organelle dynamics changes typically evolve over timescales of minutes to hours. Similar approaches can be applied to protein dynamics, such as tracking end-binding proteins that report microtubule polymerization behavior, which is highly sensitive to perturbation and can change within seconds.^92^ Cytochrome c release is another highly sensitive marker of apoptosis (Fig. 6E), and both Raman and fluorescence imaging techniques can be used to monitor cytochrome c redistribution in live cells.^63,163,164^ In addition to changes in cytochrome distribution, alterations in the redox state of cytochromes may provide another potential indicator of cellular perturbation or photodamage. Changes in cytochrome redox states can reflect disruptions in mitochondrial electron transport and cellular metabolic activity, providing complementary information to that obtained from their spatial redistribution.^165,166^ Furthermore, mitochondrial dye leakage is a common indicator of mitochondrial dysfunction, including the loss of mitochondrial membrane potential (Fig. 6E).^167,168^

At shorter timescales, ion signaling induced by femtosecond laser excitation or photothermal processes can be monitored using fluorescent sensors such as GCaMP, which enable detection of cellular responses within seconds.^169–172^ Genetically encoded voltage indicators can further detect photoperturbation-induced electrical responses on millisecond timescales in optogenetic systems.^173,174^ Although these rapid signaling responses are generally used to investigate signaling pathways and neural activity rather than to directly evaluate phototoxicity, severe photoperturbation is expected to alter these processes, such as elevated calcium responses during tissue wounding.^175–177^ Therefore, they can still be used to assess photoperturbation in biological systems.

#### 4.5.3 Oxidative stress quantification

A primary hallmark of light-induced damage is the generation of ROS. While severe oxidative stress can lead to cell death, even sublethal light exposure may disrupt intracellular calcium homeostasis or prolong mitosis.^178^ Direct ROS quantification using biosensors can be used to establish safety thresholds in SRS microscopy.^123^ More advanced longitudinal studies have combined SRS imaging with bulk RNA sequencing to perform transcriptome-wide analyses of laser exposure.^123^ These approaches can reveal the upregulation of stress-response genes, including heat shock proteins and antioxidant enzymes, providing a detailed molecular profile of laser-induced perturbation even when cells remain morphologically normal.^123^

To perform real-time ROS sensing, fluorescence-based ROS dyes such as H2DCFDA could be used (Fig. 6F).^92,179,180^ However, ROS sensors themselves may introduce perturbations to the biological system under study. Genetically encoded fluorescence ROS sensors generally produce less chemical perturbation than conventional ROS-sensitive dyes, although they require genetic modification of the cells.^181,182^

ROS generation can also induce photobleaching of fluorescent molecules, which may serve as an indirect readout of oxidative stress. Although oxygen-independent mechanisms can also contribute to photobleaching, ROS-mediated photobleaching can often be distinguished by altering oxygen levels within the cellular microenvironment.^128,183^

#### 4.5.4 Proliferation capability

Monitoring cell proliferation over time is another important longitudinal metric, as cell division is highly sensitive to perturbations before and during mitosis (Fig. 6G). For example, localized photoperturbation of centrosomes has been reported to induce abnormal daughter cell formation, indicating functionally significant disruption of cellular proliferation.^143^ In addition, proliferation rates can serve as a quantitative measure of phototoxicity (Fig. 6G). For instance, Dunnington *et al.* demonstrated that using their home-designed chamber and 40 mW pump and 50 mW Stokes power in the NIR range, imaged cells maintained proliferation rates comparable to controls, with fold increases in cell number of 1.84 ± 0.24 and 1.87 ± 0.27, respectively, over 24 hours, supporting the low phototoxicity of this imaging configuration.^155^ Such proliferation quantification is suitable not only for measuring significant photoperturbations that lead to cell death, but also for evaluating mild perturbations that induce cell senescence.

### 4.6 Common laser parameters for CRS microscopy

Many studies have evaluated the optimal laser parameters for CRS microscopy of live cells. These experimental findings provide practical value that supports the discussions in sections 4.1–4.5. For example, in an early study, Nan *et al.* differentiated the roles of peak power and average power in photodamage during CARS microscopy by independently varying pulse energy and average power.^116^ They identified a nonlinear (peak-power-dependent) damage threshold at approximately 2 nJ pulse energy (with a 711 nm pump wavelength), shown as rapid cellular boundary retraction. In contrast, a linear damage threshold was observed at 9–12 mW average power on the sample, characterized by slower but pronounced morphological changes.

In another study, Fu *et al.* investigated CARS photodamage kinetics and revealed a transition in the photodamage order with increasing peak power.^114^ In myelin samples, the photodamage rate scaled nearly linearly at lower peak powers but shifted toward a quadratic dependence at higher intensities. However, the observed “damage” in this case corresponded to late-stage severe damage induced by multiphoton ionization and avalanche effects, rather than the less catastrophic photochemical perturbations that occur during the early stages of live-cell imaging. The laser beam for perturbation is parked on the sample to accumulate damage rather than continuously scanning the sample. For live cells, they established a practical safety threshold in which continuous scanning at a total average power of 2.3 mW (7.8 MHz) caused no observable damage over 10 minutes, whereas 9.24 mW induced membrane blebbing after only 1 minute of continuous imaging.^114^

As discussed in Section 4.3, higher pump power is generally more effective for signal generation in CARS imaging and is therefore preferred. In SRS imaging, however, the photodiode saturation limit places an upper bound on the power of the detected beam. Consequently, the Stokes beam is typically operated at a higher power and intensity-modulated, while the pump beam is detected, because the longer-wavelength Stokes beam generally poses a lower risk of photodamage. In SRS microscopy, intensity modulation is commonly implemented using sinusoidal or square-wave modulation at frequencies below the laser repetition rate but above 1 MHz to minimize laser intensity 1/*f* noise.

Commercial oscillators commonly used for CRS microscopy typically operate at a fixed repetition rate of approximately 80 MHz. While this high repetition rate enables rapid imaging, it may not be optimal for minimizing photodamage in CRS microscopy. The effective repetition rate can be reduced through pulse-picking approaches using either a Pockels cell or an AOM, provided the laser pulse energy is sufficiently high.^116,136^ Fu *et al.* investigated this relationship in CARS microscopy using a Pockels cell to vary the repetition rate and found that lower-order photodamage processes dominated above 0.975 MHz.^114^ Similarly, Clark *et al.* demonstrated that pulse picking with lower duty cycles (corresponding to repetition rates of 1–8 MHz) improved SNR and reduced phototoxicity at the same average input power.^136^

CRS microscopy is conventionally performed using NIR pulsed laser excitation to minimize phototoxicity, where nonlinear photodamage is typically the dominant mechanism.^103,119^ The sample DT could be affected by the wavelength used for imaging, even in the NIR range. For example, Fu *et al.* reported that, in CARS imaging, excitation wavelengths below 760 nm caused myelin damage within one minute, whereas wavelengths between 780 and 880 nm produced no observable photodamage over five minutes.^114^ The reported damage corresponded primarily to multiphoton ionization and avalanche effects, which occur at power levels far beyond the tolerance of most live-cell imaging conditions. In live-cell SRS imaging, Stiebing *et al.* demonstrated that 785 nm excitation minimized photodamage and enabled stable imaging at 60 mW average power, whereas excitation wavelengths shorter than 785 nm resulted in cell death within one hour.^184^ Another study using femtosecond-pulse SRS microscopy quantified photodamage thresholds based on cellular morphology changes and found that the critical power density increased substantially with wavelength, from 1.6 MW cm^−2^ at 680 nm to 15.8 MW cm^−2^ at 1000 nm.^142^

When visible pulses are used for imaging, higher spatial resolution can be achieved. However, phototoxicity increases substantially due to both stronger linear absorption and enhanced multiphoton absorption efficiency. Photoionization is much more likely to occur with visible pulsed excitation, although this effect can be mitigated by extensive pulse chirping. Importantly, the absence of photoionization or avalanche effects does not necessarily indicate negligible photoperturbation to the sample. For longitudinal live-cell imaging, additional metrics for evaluating subtle phototoxic effects, as discussed in section 4.5, must be considered. Given the relatively strong photoperturbation associated with visible excitation and the SNR requirements of CRS microscopy, visible wavelengths are generally not preferred for longitudinal live-cell CRS imaging.

Nonlinear photodamage in CRS microscopy can also be mitigated by employing longer pulse widths, which reduces peak power while maintaining the same average power. This strategy, however, reduces signal level in CRS compared to using shorter pulses. Koike *et al.* demonstrated that 7 ps and 14 ps pulses allowed higher permissible average powers for live-cell SRS imaging at fixed SNR compared with more damaging 2 ps pulses.^185^ Similarly, pulse chirping effectively reduces severe nonlinear photodamage in visible-SRS imaging by lowering the peak power. Lin *et al.* reported a tenfold increase in the photodamage threshold by chirping pulses from 0.15 ps to 4 ps in visible-SRS microscopy.^119^

It is also important to note that although NIR laser pulses generally exhibit much lower multiphoton absorption than visible pulses, water overtone absorption bands, particularly around 1450 nm, should be avoided because of their strong photothermal effects. If either the pump or Stokes beam overlaps with these wavelengths, substantial photothermal perturbation may occur, potentially altering cellular functions during longitudinal imaging experiments.

Table 4 summarizes representative laser parameters used in CRS microscopy for unlabeled live-cell imaging. It is important to note that, for longitudinal live-cell imaging of the same cells, photoperturbation should be minimized as much as possible. Variations in the reported threshold values among different studies likely arise from inconsistencies in experimental parameters, including pixel dwell time, image size, sample type, and imaging system optimization conditions, and inconsistencies in evaluating phototoxicity.

**Table 4:** Representative laser parameters used in CRS microscopy for live-cell imaging

Sample type	Average power (mW)	Excitation wavelength (nm)	Pulse width	Repetition rate (MHz)	Ref.
SKOV3 cells	Stokes: 7.5 pump: 15	1045, 805	∼3–4 ps	80	186
Dividing mammalian cells	Stokes: 40 pump: 30	1064, 812	∼7 ps	80	187
*E. coli*	Stokes: 12 pump: 15	1040, 800	∼2 ps	80	188

## 5. Longitudinal live-cell CRS microscopy

In this section, we briefly overview examples of live-cell CRS microscopy, with particular emphasis on longitudinal live-cell imaging. Here, longitudinal imaging refers to repeated imaging of the same cells over an extended period to monitor dynamic changes within the same group of cells.

### 5.1 Temporal sampling across parallel cell populations

**Fig. 7:**
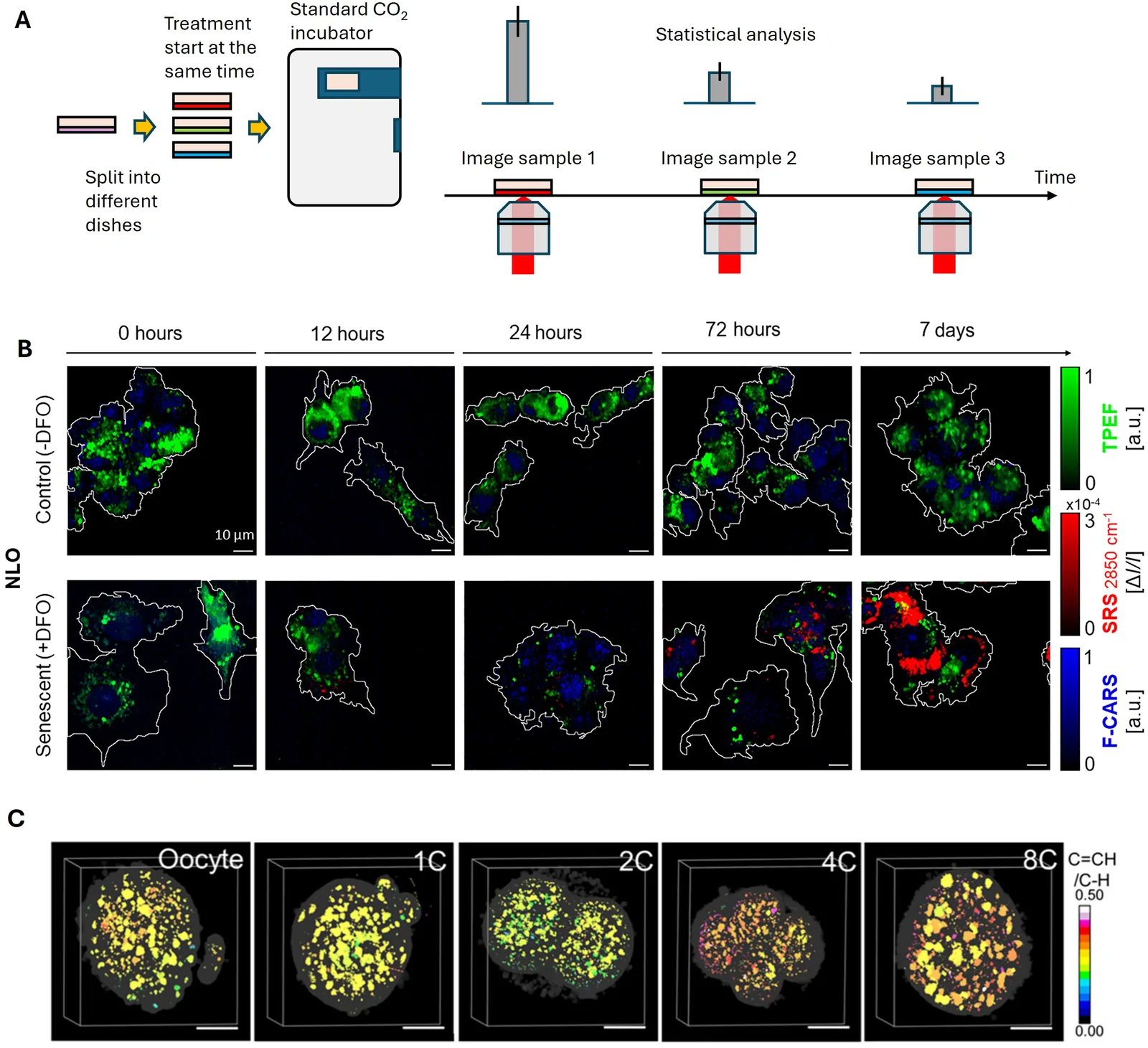
Temporal sampling across parallel cell populations. (A) Schematic illustration of temporal sampling from parallel cell populations cultured in separate dishes. Scientific conclusions are derived from statistical analyses of phenotypic and chemotypic traits across cell populations. (B) Time-dependent metabolic changes measured by nonlinear optical (NLO) microscopy. Autofluorescence signals acquired by two-photon excited fluorescence (TPEF) are shown in green, lipid droplet (LD) signals detected by SRS microscopy are shown in red, and nuclei are visualized by forward CARS (F-CARS). The top row represents the control group, whereas the bottom row shows senescent cells after being treated with 100 μM deferoxamine (DFO). Figure panel adapted from ref. 196. (C) Lipid saturation levels (C=C–H/C–H intensity ratio) in LDs during early mouse embryonic development at different stages, revealed by hyperspectral three-dimensional SRS microscopy. Figure panel adapted from ref. 190. Scale bars: 20 μm for panel C.

It is worth noting that most biological studies using CRS microscopy were not performed in a longitudinal manner, which typically requires incubation chambers integrated with the microscope and precise control of the cellular microenvironment over time scales ranging from hours to days. In contrast, when tracking temporal changes in the same cells is not required, and statistical readouts are sufficient to draw conclusions, experiments can be performed by imaging separate populations (or dishes) of cells at different time points (Fig. 7A). In such cases, cells may either be fixed to preserve defined time points or imaged live within a short acquisition window. Statistically meaningful information can still be obtained when the observed biochemical changes are sufficiently large and cellular metabolism is relatively homogeneous across the population.

This temporal sampling across parallel cell populations is one of the most widely used approaches in Raman and CRS microscopy for assessing cellular chemical changes over time. This strategy is applicable across diverse systems, ranging from bacterial cells^153^ to 2D mammalian cell cultures,^9,154,189^ to 3D oocytes,^190^ and to more complex tissues such as the *Drosophila* ovary^191^ and *Caenorhabditis elegans* (*C. elegans*).^192^ When combined with isotope labeling or Raman tags, it can further resolve metabolic activity, particularly biomass synthesis, by distinguishing newly synthesized molecules from pre-existing ones.^37,83,153,154,189,193,194^ Similarly, drug or other compound uptake and intracellular accumulation can also be monitored using this approach.^61,195^Fig. 7B shows the changes in NADH, LDs, and nucleic acids measured by TPEF, SRS, and forward CARS modalities, respectively, for cells in the control and the senescent groups.^196^ Images are acquired by temporal sampling using parallel cell populations. The accumulation of LDs in the senescent cells was detected over 72 h. Fig. 7C presents an example of changes in lipid saturation levels during early embryonic development, from the oocyte to the 8-cell stage, quantified by the ratio of the C=C to C–H Raman peak intensities obtained from hyperspectral SRS imaging.^190^

A key advantage of this population-based temporal imaging strategy is that samples do not need to be maintained alive or minimally perturbed after imaging. As a result, sample preparation and imaging workflows are generally simpler and less demanding. However, a major limitation is that temporal changes are not measured within the same cellular population; therefore, dynamic chemical trajectories at the single-cell level cannot be resolved. Instead, the analysis relies on statistical differences between populations at different time points, which may be influenced by intrinsic heterogeneity or subpopulations with distinct chemotypic features within the sample.

### 5.2 Longitudinal live-cell imaging

For longitudinal imaging of live mammalian cells, in addition to minimizing phototoxicity, it is essential to maintain a stable and physiologically relevant microenvironment for the cultured cells. For most 2D mammalian cell cultures, this requires: (1) maintaining the sample at physiological temperature; (2) maintaining 90–95% humidity to minimize medium evaporation; (3) maintaining 5% CO_2_ to preserve proper medium pH; (4) providing appropriate O_2_ levels to support cellular respiration; and (5) preventing contamination arising from prolonged exposure to the external environment. These conditions can be achieved by placing cell culture dishes, with their lids kept closed, inside a microscope incubation chamber or stage-top incubator. Such environmental control systems are critical for longitudinal monitoring of the same cells over extended periods.

In addition, maintaining positional and focal stability is important for longitudinal imaging of the same FOV. Motorized sample stages combined with automated stage registration software can be used to maintain the sample positioning throughout the longitudinal imaging period. Focus drift can be compensated for by autofocusing systems or manually adjusted when necessary. For experiments running longer than a few hours, air- or oil-immersion objectives are generally preferred over water-dipping objectives, as they avoid losing focus caused by evaporation of the immersion water during prolonged imaging sessions.

**Fig. 8:**
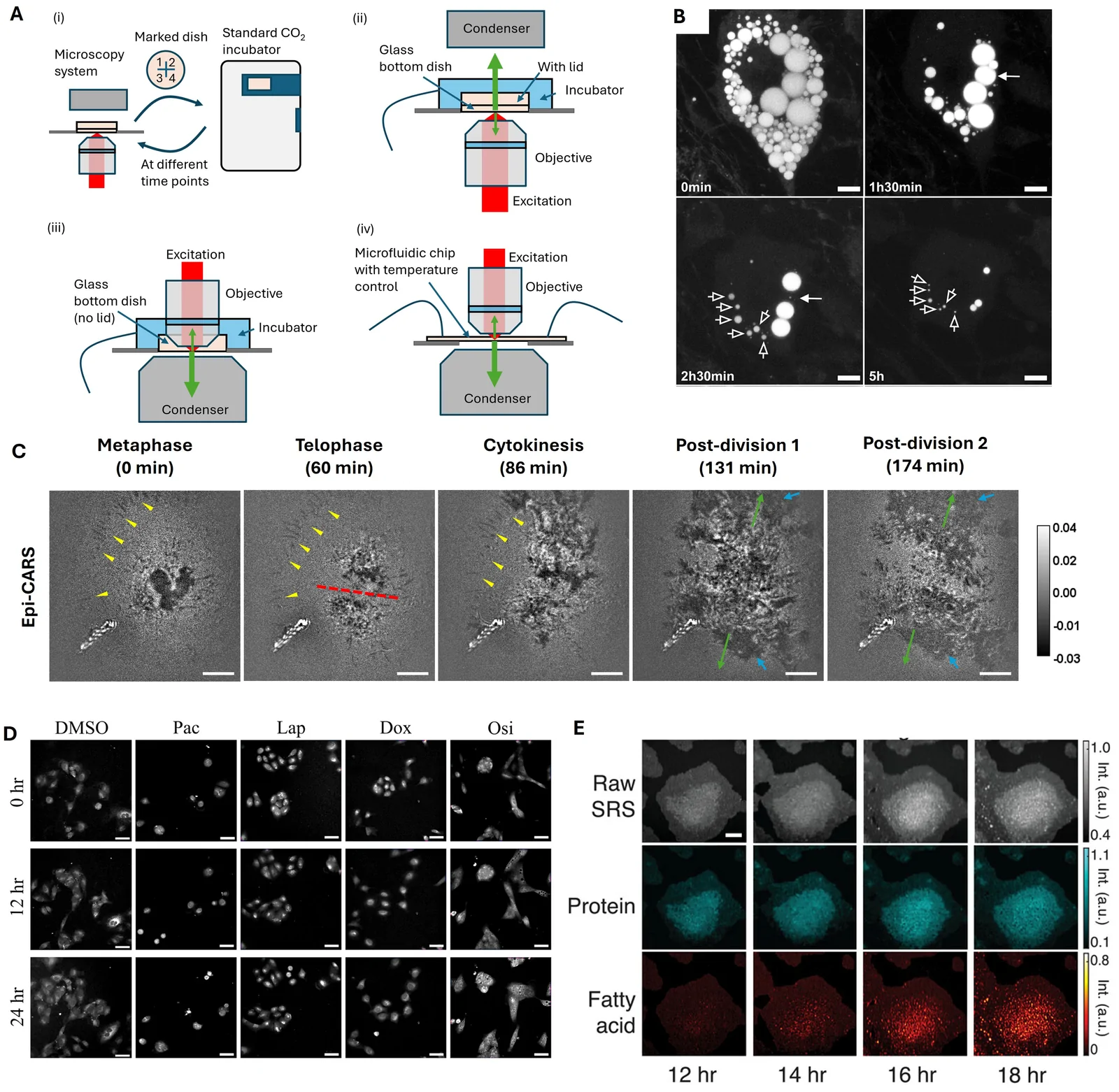
Longitudinal CRS microscopy. (A) Configurations used for longitudinal CRS microscopy. (i) Cells imaged sequentially and returned to a conventional cell incubator between imaging sessions. The culture dish is marked to enable relocation of the same field of view. (ii) Cells cultured in a dish and maintained in a stage-top incubator for imaging with an inverted microscope configuration. (iii) Cells cultured in a dish and maintained in a stage-top incubator for imaging with an upright microscope configuration. (iv) Cells cultured in a microfluidic chip for longitudinal imaging. (B) Longitudinal CARS imaging showing lipid droplet degradation over time in 3T3-L1 cells. Figure panel adapted from ref. 198. (C) Longitudinal *epi*-CARS imaging showing cell adhesion dynamics (negative contrasts) during mitosis and post-division migration. Yellow arrows indicate filopodia adhesion sites. Blue arrows indicate newly formed adhesion areas after cell division. Green arrows denote the migration directions, and the red dashed line marks the division center. Figure panel adapted from ref. 201. (D) Time-lapse SRS imaging of A549 cells in the control group and following treatment with different compounds. Pac: paclitaxel; Lap: lapatinib; Dox: doxorubicin; Osi: osimertinib. Figure panel adapted from ref. 155. (E) Time-lapse SRS imaging showing protein and fatty acid synthesis in engineered *E. coli* cells. Figure panel adapted from ref. 188. Scale bars: 10 μm for panels B and E, 5 μm for panel C, and 20 μm for panel D.

Alternatively, if the image sessions are brief and do not impose substantial environmental stress on the samples, cells can be periodically imaged and then returned to a conventional cell culture incubator between imaging sessions (Fig. 8A(i)). This approach may not require the use of incubation systems attached to the microscope. However, it requires careful marking of the culture dish, as relocating and co-registering the same FOV, particularly over extended periods, can be extremely challenging and time-consuming. Tracking the same cells over long durations with this approach could also be difficult because of cell migration, proliferation, and changes in morphology. Di Napoli *et al.* applied hyperspectral CARS microscopy together with this approach to quantify LD amount and content changes during adipose stem cell differentiation.^197^ The results revealed that lipid uptake during adipogenic differentiation is highly heterogeneous among individual LDs and depends on droplet size, time, and fatty acid composition. The study further showed that exogenous fatty acids are incorporated into LDs with distinct kinetics, with cells exhibiting faster uptake during early differentiation than after differentiation has progressed, indicating dynamic regulation of lipid metabolism over time.^197^

Configuration-wise, inverted microscopes are generally better suited for integration with stage-top incubators because they provide greater working space and allow imaging through the bottom of culture dishes without opening the lid (Fig. 8A(ii)). This configuration is compatible with CARS microscopy in both forward and *epi*-detection geometries. However, for SRS microscopy, the upright microscope configuration (Fig. 8A(iii)) is often preferred for cell imaging, as the SRS signals from transparent samples such as cells predominantly propagate in the forward direction. In addition, SRS detection typically requires a condenser with a higher NA than the excitation objective to avoid the contribution from cross-phase modulation (XPM). High-NA oil condensers are commonly used in upright systems but are less readily available or practical in inverted configurations. When SRS is implemented on an inverted microscope, the conventional low-NA condenser is often insufficient, and a high-NA objective placed above the sample is required for efficient photon collection, which in turn compromises working space. Although most stage-top incubators are designed for inverted microscopes, specialized versions are available for upright systems. Yuan *et al.* developed a dedicated and flexible incubation chamber for longitudinal SRS microscopy and demonstrated stable imaging of the same FOV for 24 hours.^186^ In this configuration, because the cell culture dish lid must be removed during imaging, all components require careful disinfection and reassembly, making the workflow substantially more complex than standard stage-top incubator systems used in inverted fluorescence microscopy. As a result, such configurations are less practical for routine biological applications.

For CARS microscopy, the configuration constraints are considerably relaxed because CARS does not suffer from the potential XPM background. Standard stage-top incubators used with inverted microscopes are therefore compatible with CARS imaging in both forward and *epi*-detection modes. One potential limitation in forward detection is water condensation on the incubator or dish lid, which can degrade collimation and reduce the transmission efficiency of forward-propagating CARS signals. This typically occurs when the stage-top incubator is operated in isolation without a larger environmental enclosure that minimizes temperature difference across the lid. Such condensation effects generally do not impact *epi*-detected CARS measurements.

Forward-detected CARS has been employed for longitudinal imaging of lipid metabolism. For example, Paar *et al.* used forward CARS microscopy to monitor LD degradation in live 3T3-L1 adipocytes stimulated with forskolin to induce lipolysis, enabling visualization of substantial LD degradation within the same cells over time (Fig. 8B).^198^ Takei *et al.* studied brown adipocyte lipid metabolism using multiplex CARS microscopy and traced the same group of cells for up to 9 h using an onstage incubator.^199^ Using the ratios of C–D/C=O and C=C/C=O, it was concluded that HB2 cells exhibited more resistance to lipid saturation compared with 3T3-L1 cells. Longitudinal *epi*-CARS imaging was also adopted to monitor changes in LD distribution under hypoxic conditions,^200^ revealing the accumulation of LDs in the endoplasmic reticulum during prolonged hypoxia exposure. Furthermore, Zhou *et al.* reported that *epi*-CARS can generate unique negative contrast associated with cell–substrate adhesion dynamics occurring within tens of nanometers from the substrate surface.^201^ When implemented in a confocal configuration with a pinhole positioned at the plane conjugate to the laser focus, out-of-focus *epi*-CARS reflections can be effectively suppressed, thereby enhancing interface-specific contrast. Using this approach, longitudinal *epi*-CARS microscopy revealed both the division of adhesion sites during mitosis and the continuous outward expansion of homogeneous adhesion regions (Fig. 8C).^201^

Besides using conventional glass-bottom cell culture dishes, an alternative approach is developing and applying microfluidic culture chambers, which offer a much thinner geometry and are therefore compatible with both upright and inverted microscope configurations (Fig. 8A(iv)). Such platforms are suitable for both CARS and SRS imaging, provided that the chamber materials are carefully selected. In particular, glass- or quartz-based chambers are preferred to avoid strong vibrational backgrounds commonly associated with polymer-based materials. Compared with conventional culture in glass-bottom dishes, microfluidic platforms are less widely adopted due to higher system complexity and cost. However, they offer several important advantages, including (1) continuous renewal of culture medium *via* controlled flow and pumping, (2) precise temporal control of nutrient composition and drug treatments, enabling sequential perturbation of the same cell population, and (3) the ability to study cellular responses under physiologically relevant flow conditions. For example, Tipping *et al.* utilized SRS microscopy and a perfusion chamber to visualize the real-time uptake of the alkyne-tagged drug 7RH in live cancer cells. The approach successfully tracks drug accumulation, evaluates combination treatment with cisplatin, and provides a novel readout for monitoring inhibited cell migration in breast cancer models.^202^ In another study, the time-dependent intracellular accumulation of two other alkyne-tagged drugs was monitored in living cells for over 60 minutes.^203^ Using a microfluidic platform integrated with live-cell SRS imaging, Cao *et al.* quantified LD dynamics in thousands of individual cells exposed to multiple chemical conditions and revealed substantial cell-to-cell heterogeneity in LD responses.^204^ Their results showed that free-fatty-acid-induced lipid accumulation in nonadipocyte cells primarily occurred through an increase in LD number rather than changes in droplet size or lipid concentration.^204^ In another study, Dunnington *et al.* developed a 3D-printed perfusion platform for SRS microscopy. It achieved real-time longitudinal imaging of live cells with minimal phototoxicity.^155^ By extracting chemical and morphological features over 24 hour time-lapses, the system successfully tracks heterogeneous single-cell trajectories and drug responses (Fig. 8D).^155^

When imaging cells within live organisms, such as small animals, many of the physiological conditions described above are naturally maintained by the organism. In these cases, environmental control is less critical, whereas sample motion and tissue thickness introduce additional imaging challenges. For example, fs SRS imaging was applied in live early *Drosophila* embryos to understand LD dynamics during embryo development.^205^ It measured the speed and turning frequency of LDs at different apical-to-basal depths and developmental stages and then used these to interpret changes in droplet density distribution throughout the embryo. The results show that the position dependence of the velocity-reversing rate is the critical regulatory point that makes the switch from phase II (basal accumulation) to phase III (apical redistribution).

Furthermore, bacterial cells, fungal cells, and plant cells can also be maintained at room temperature without the need for precise microenvironment control. Tague *et al.* applied SRS microscopy as a label-free approach to monitor the production of free fatty acids in engineered *Escherichia coli* (*E. coli*) at single-cell resolution over several cell cycles (Fig. 8E).^188^ This approach allowed for the in-depth analysis of production dynamics and heterogeneity in growing microcolonies. In summary, the findings of the study indicated that longitudinal hyperspectral SRS can trace engineered microbial cells over multiple generations while monitoring product accumulation, composition, and heterogeneity at single-cell resolution. In fatty-acid-producing *E. coli*, production variability emerges across colonies and among cells within the same colony rather than existing only as a bulk population property. Bae *et al.* used hyperspectral SRS microscopy with an aryl-alkyne Raman-tagged vancomycin to dynamically image its uptake and distribution within *Staphylococcus aureus* biofilms over time, revealing non-uniform diffusion and preferential binding of the antibiotic to bacterial clusters.^206^ Furthermore, the growth and proliferation of yeast typically do not require strict environmental conditions such as 5% CO_2_, 90–95% humidity, and 37 °C physiological temperature. Okuno *et al.* reported using broadband hyperspectral CARS microscopy to image chemical distribution changes during yeast budding.^207^

## 6. Future perspectives

As CRS microscopy has matured into a powerful tool for studying cellular metabolism, longitudinal imaging of live cells is becoming increasingly feasible and adopted. Ultimately, the impact and prevalence of this approach will depend on how much information it can provide beyond that obtainable through conventional methods, in which cells are fixed or transiently imaged at discrete time points and phenotypic or chemotypic changes are analyzed statistically across parallel populations. As discussed in sections 2 and 3, the applications of label-free CRS microscopy are largely constrained by its LOD and chemical selectivity. Consequently, CRS is most effective for imaging highly enriched chemical species in biological samples, such as LDs, hydrophobic drug compounds, and retinoids. Although chemical labeling strategies can substantially improve chemical selectivity, the LOD of vibrational imaging remains much higher (inferior) than that of fluorescence microscopy because of the intrinsically higher background in aqueous environments. Furthermore, genetically encoded Raman tags are currently far less feasible and robust than fluorescent protein-based labeling approaches.

For imaging highly enriched compounds, longitudinal CRS microscopy has already demonstrated significant capabilities in monitoring changes in LD abundance, composition, and dynamics. However, quantitative analysis of lipid composition generally requires multichannel or hyperspectral imaging. In live-cell applications, conventional frame-by-frame hyperspectral acquisition is often unsuitable because LD motion during image acquisition can distort the reconstructed spectra. Instead, line-by-line hyperspectral imaging or multiplex CRS approaches based on broadband detection are preferred.^208–212^ The spectrum needs to be acquired on the millisecond or microsecond timescale, so that motion artifacts do not significantly impact the organelle spectrum. Although substantial technical progress has been made in these areas, many implementations remain at the proof-of-concept stage or have primarily been applied to fixed cells and tissues rather than longitudinal live-cell imaging. In addition, these advanced hyperspectral methods often require complex instrumentation, high laser powers, and elevated pulse energies. The resulting phototoxicity and photoperturbation have not yet been systematically characterized, and their suitability for long-term live-cell imaging remains uncertain. Broadband CRS approaches may introduce high phototoxicity because achieving adequate signal levels over a broad spectral range often requires higher total laser power and pulse energy than narrowband implementations. Broadband CARS has been reported to image cells separately collected at different division phases,^213^ but has not yet demonstrated following the same cell through the entire division process. The femtosecond laser pulse potential casts a strong optical perturbation on the dividing cells and would likely halt the mitosis. If the laser beam is focused on the interfacial region, femtosecond *epi*-CARS can track the dynamic changes of cell adhesion throughout and post-mitosis.^201^ Picosecond laser pulses, on the other hand, have much lower phototoxicity and were reported to track the entire cell mitotic process.^36,187^

To date, most longitudinal CRS studies have been performed using cells cultured in 2D environments, which represent the most widely applied and experimentally straightforward model system. In contrast, CRS imaging of 3D cultures, spheroids, and organoids has been extensively explored for fixed samples but has rarely been applied to longitudinal live-cell imaging. This limitation largely reflects the additional technical challenges associated with maintaining and imaging living 3D systems. As sample thickness increases, light penetration becomes a major obstacle to visualizing deeper layers. Although numerous tissue-clearing methods have been developed, they are incompatible with live-cell imaging.

Optofluidic platforms offer an attractive solution for integrating live-cell culture, environmental control, and optical imaging. Several groups have demonstrated optofluidic systems for cell imaging and flow cytometry analysis.^155,204^ Compared with conventional culture dishes, microfluidic platforms require more sophisticated device fabrication and sample handling but provide substantially greater control over the cellular microenvironment. Such systems are particularly well-suited for advanced cell culture models, including stem cell-derived organoids and other complex biological systems.^214,215^

Beyond imaging cells cultured under 2D or 3D conditions, studies in complex live organisms can provide a more physiologically relevant and integrated model system. However, because optical imaging techniques generally exhibit limited penetration depth in nontransparent tissues, most investigations are restricted to surface regions unless surgical exposure is performed. In contrast, optically transparent organisms, such as zebrafish,^216^*Drosophila melanogaster*,^194,205,217^ and *C. elegans*,^218–220^ offer excellent platforms for monitoring biochemical processes in deeper tissue layers.

Furthermore, CRS microscopy has been applied predominantly in studies of mammalian cells, whereas its use in live plant cell research is less explored in comparison. Long-term imaging of metabolic dynamics in plant cells could provide valuable insights into plant physiology and cellular metabolism.^221–223^ Imaging plant cells containing chloroplasts presents challenges, as chlorophyll absorption and autofluorescence generate substantial background signals in SRS and CARS modalities, respectively.

Longitudinal imaging of the same group of live cells is especially valuable for understanding how sustained stressors influence cellular function over time. Characterizing the progressive responses of cells to environmental, chemical, or physical stress requires repeated imaging of the same FOV over extended periods. In this context, CRS microscopy is uniquely positioned to monitor metabolic changes, particularly alterations in lipid metabolism and biomass production. To accurately interpret stressor-induced effects, however, it is essential to ensure that neither the excitation lasers nor the cell environment introduce metabolic changes that could obscure the biological processes under investigation.

A promising direction involves understanding how localized perturbations produce both local and system-wide cellular responses. Such studies could provide new insights into spatial biochemistry and intracellular signaling. Conventional chemical treatments generally lack spatial confinement because freely diffusing molecules interact broadly throughout the sample. Optical approaches offer significantly improved spatial precision. For example, optogenetic techniques enable light-controlled regulation of neuronal activity and have transformed the study of neural circuits.^224–226^ Similarly, femtosecond laser excitation has been shown to induce localized cellular responses such as calcium elevations.^169,170^ More recently, real-time precision opto-control (RPOC) has enabled localized generation of ROS and activation of photoresponsive compounds within selected subcellular regions or individual cells.^128,143,167,227^ Combining these perturbation methods with CRS imaging as a metabolic readout may establish new paradigms for studying cellular responses to spatially controlled stimuli.

Wide-field imaging platforms for long-term live-cell studies are comparatively mature and have been extensively commercialized. In contrast, laser-scanning approaches, including both CRS and confocal fluorescence microscopy, are less commonly used for longitudinal imaging applications, especially for multiple FOVs. To repeatedly image the same FOV over extended periods, these systems must accurately record and revisit multiple spatial locations at user-defined time intervals. Moreover, because confocal fluorescence and CRS microscopy typically image only a thin optical section, axial sample drift occurring either during long-term imaging or while switching among multiple FOVs can significantly affect image quality and reproducibility. Robust strategies for automated focus stabilization and drift correction will therefore be critical for the future development of longitudinal live-cell CRS imaging platforms.^228^

## 7. Conclusions

In this review, we focus on longitudinal live-cell CRS imaging. We first discuss the LOD of CRS microscopy, followed by a comparison of the chemical selectivity of CRS and fluorescence microscopy. We then examine the origins and mechanisms of phototoxicity and photoperturbation in biological imaging, develop a mathematical framework to describe photoperturbation under different laser configurations, and review metrics for evaluating phototoxicity and photoperturbation in live cells. Next, we highlight recent approaches in live-cell CRS microscopy and their applications. Finally, we discuss future perspectives and emerging directions for longitudinal live-cell CRS imaging. As CRS technology continues to mature and expand in capability, longitudinal imaging of the same cell populations is expected to provide new insights into dynamic cellular chemistry and responses to environmental and physiological stressors that cannot be captured by conventional static or transient imaging approaches. Better exploring this potential will require improved control of the cellular microenvironment during imaging, as well as a deeper understanding of phototoxicity and photoperturbation mechanisms. Ultimately, the impact of CRS microscopy on cell biology is largely determined by its LOD and chemical selectivity in complex biological environments.

## Author contributions

All authors contributed to the writing of this article.

## Conflicts of interest

Dr Chi Zhang is the founder of Photokinesis LLC, a startup dedicated to commercializing advanced optical control technologies. Other authors declare no conflicts of interest.

## Data Availability

No primary research results, software, or code have been included, and no new data were generated or analysed as part of this review.
